# Interspecific Comparison of Orthologous Short Interspersed Elements Loci Using Whole-Genome Data

**DOI:** 10.3390/genes14112089

**Published:** 2023-11-17

**Authors:** Sergei Kosushkin, Vitaly Korchagin, Andrey Vergun, Alexey Ryskov

**Affiliations:** 1 Laboratory of Genome Organization, Institute of Gene Biology of the Russian Academy of Sciences, Vavilova Str., 34/5, Moscow 119334, Russia; vitaly_korchagin@rambler.ru (V.K.);; 2 Department of Biochemistry, Molecular Biology and Genetics, Moscow Pedagogical State University, 1/1 M. Pirogovskaya Str., Moscow 119991, Russia

**Keywords:** SINE, orthologous loci, polymorphism, evolution, reptiles, comparative genomics

## Abstract

The polymorphism of SINE-containing loci reflects the evolutionary processes that occurred both during the period before the divergence of the taxa and after it. Orthologous loci containing SINE in two or more genomes indicate the relatedness of the taxa, while different copies may have a specific set of mutations and degree of difference. Polymorphic insertion can be interpreted with a high degree of confidence as a shared derived character in the phylogenetic reconstruction of the history of the taxon. The computational comparison of the entire set of SINE-containing loci between genomes is a challenging task, and we propose to consider it in detail using the genomes of representatives of squamate reptiles (lizards) as an example. Our approach allows us to extract copies of SINE from the genomes, find pairwise orthologous loci by using flanking genomic sequences, and analyze the resulting sets of loci for the presence or absence of SINE, the degree of similarity of the flanks, and the similarity of the SINE themselves. The workflow we propose allows us to efficiently extract and analyze orthologous SINE loci for the downstream analysis, as shown in our comparison of species- and genus-level taxa in lacertid lizards.

## 1. Introduction

Short interspersed elements (SINEs) form a significant part of the genomes of many animals [1,2]. Within a single genome, there may be several families of SINEs, which in turn can be divided into subfamilies due to the process of accumulation of specific mutations and subsequent waves of amplification [3]. The value of orthologous SINE insertions for phylogenetic comparisons is due to their relatively well-studied life cycle, for which a homoplasy, including cases of precise removal of the element from the site of genomic integration, is rare [4,5,6]. The discovery of such loci, therefore, serves as a source of rich and highly reliable evolutionary signals [7,8]. At the same time, it is informative to compare orthologous loci in terms of the presence/absence of SINE in a certain locus [9], as well as to compare individual mutations between already inserted SINEs [10].

The reliable identification of orthologous SINE loci is a significant challenge for a number of reasons. Copies sometimes number in the tens and hundreds of thousands, which increases the computational complexity and the probability of chance matches and abnormal false-positive and false-negative signals. The quality of the genome assembly and its comparability between the taxa studied have a significant impact. The task is complicated by the fact that sometimes it is not convenient and justified to make pairwise or multiple whole-genome alignments for all genomes as suggested by other approaches [11,12,13]. Most methods for detecting retrotransposon insertion polymorphism on an intraspecific and intrageneric level are based on the analysis of short reads utilizing a reference genome [14,15,16,17]. Another common approach is to establish orthology by searching for similar flanks adjacent to the SINE in at least one of the compared assembled genomes [18], but it also has a number of objective difficulties. Such methods allow multiple valid data to be obtained and analyzed in the context of the genomic environment. However, the genome alignment step itself contains technical difficulties due to the significant computational resources required. In addition, in case of low-quality genomic assemblies, e.g., those based on short reads, the alignment becomes even more difficult, which particularly affects regions enriched with repeats. Our proposed approach utilizes widely available standard bioinformatics tools and allows for flexible parameter tuning at different stages, which can be critical for the output data. Our analysis takes several hours to complete, and its intermediate results allow us to make changes to the process at any time. In addition, our workflow addresses approaches to difficult cases of multimapping loci flanked by similarity to more than one locus in the target genome.

Here, we propose a methodology developed by us for the search and analysis of orthologous SINE insertions using the example of full-genome assemblies of four species of lizards from the Lacertidae family, the genome of one of which was assembled and published by us earlier [19]. The search for orthologs is accompanied by the filtering of results and their automatic analysis of similarity of various parts of compared sequences.

Squam1 SINE (initially called SAURIA in [20]) is widespread in the genomes of squamate reptiles, reaching hundreds of thousands of copies. We use the name Squam1 instead of SAURIA, since Sauria is the name of a taxon and should not be used as the name of a SINE family. We have previously shown the presence of a large number of copies of this SINE family in the genomes of lacertids, including young copies, so it is convenient for intergenomic analysis [21]. As an object, four species of lacertid lizards—two wall lizards *Podarcis* (*Podarcis raffonei* and *P. muralis*), one Caucasian rock lizard (*Darevskia valentini*), and a sand lizard (*Lacerta agilis*) that differs from the others ecologically, not living on rocks and cliffs—were chosen. These genera split around 43–48 MYA according to the TimeTree estimate, and *Podarcis* species split around 12 MYA [22]. We provide an effective and detailed workflow for the detection, pairwise or multiple comparison, and subsequent analysis of orthologous SINE-containing loci; discuss the difficulties that arise; and suggest possible ways to further develop the search and analysis techniques. Our methodology can be widely used for any SINE-containing genomes, as well as other multicopy interspersed repeats.

## 2. Materials and Methods

**Figure 1:**
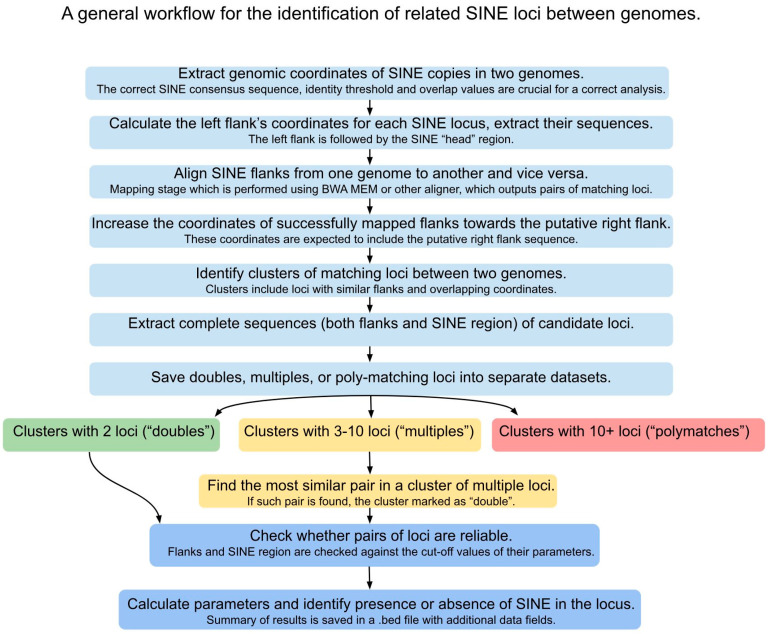
A brief overview of the analysis steps.

For analysis (Figure 1), complete genomic assemblies of lizards from the Lacertidae family deposited in NCBI genomes database were selected: *Lacerta agilis* (rLacAgi1), *Darevskia valentini* (Dval_245), *Podarcis raffonei* (rPodRaf1), and *Podarcis muralis* (PodMur_1.0 and LU_Pmuni_1.1). Abbreviated names used in the text are lag, dva, pra, and pmu (for PodMur_1.0).

In each genome, copies of the SINE Squam1 were found using the ssearch36 program from the 3.6 release of the FASTA package [23] with the use of a custom script that allows for repeated search until no new findings are made. The selection criteria were 65% similarity and 80% of the consensus length. The Squam1 consensus was determined based on the multiple alignment of randomly selected copies from the *D. valentini* genome (consensus sequence and alignment of individual loci are provided in the Supplementary Data).

The following steps were performed using a custom script available at https://github.com/Toki-bio/SINE_orth_loc/, accessed on 1 October 2023. Copies located closer than 300 bp to each other were excluded from the analysis. For each copy, the left (relative to SINE) 300 bp flank was extracted using flank from the bedtools package [24]. The flanks were mapped to the target genome in both directions using BWA-MEM [25] with default parameters. The resulting SAM file was converted to BED format with preservation of the target genome coordinates using sam2bed tool [26]; mapped sequences shorter than 100 bp were excluded. All four sets of coordinates in both BED files were increased to the right by the length of SINE + 300 bp using bedtools slop, so that the resulting interval would overlap the possible SINE insertion and the right flank. The results of both mappings were combined into one file, and for each genome, clusters of loci with overlapping coordinates were identified. The results of clustering for each species, after sorting by genomic coordinates, were merged using bedtools merge with the preservation of the original cluster information. Pairwise linked clusters were combined into groups with connected similarity. The corresponding genomic sequences were extracted from each cluster using bedtools getfasta and seqkit [27]. Based on the number of loci in the cluster, we divided them into double, multiple (3–10 loci), and poly-matching loci (>10 loci).

Doubles were directly analyzed with a custom script for their quality and properties (reliable flanks and a SINE insertion). In short, the triple alignment of two loci from different genomes and the Squam1 consensus was divided into three parts (two flanks and SINE), for each of which the length and degree of similarity (using esl-alipid from HMMER package, http://hmmer.org/) was calculated, and for SINE, its presence or absence and the degree of similarity both between genomes and with the consensus were taken into account. If the length of the left or right flank was less than 150 nt or if there was a SINE at the right or at the left end of the alignment, the locus was discarded. The identity threshold for left and right flank was set to 65%. For the putative SINE-containing region, the cut-off was set at 100 nt length and 65% identity. In all steps, manual inspection of positive and negative results allowed for the optimization of the filtering conditions. Validated loci received a status of “plus-plus”, “plus-minus” or “minus-plus”.

Clusters containing multiple loci were analyzed for finding the closest pair of sequences from two different genomes. Thereafter, the quality of the locus was determined as described above for double loci, after which the selected clusters were filtered for sufficient similarity of the remaining sequences, which made it possible to collect reliably similar loci in the cluster. If, after selection, there remained two loci from different species, then such clusters were combined with doubles and analyzed further on an equal basis with them.

The output final file contained information about pairs of orthologous loci, the presence of Squam1 in them, and numerical alignment properties. The following parameters were calculated and saved in a final output file for each alignment of a particular locus and SINE consensus: in left or right flanks, it was the number of identical nucleotides and pairwise identity; in the SINE region, it was pairwise identity and the number of identical nucleotides between genomes, pairwise identity, and number of identical nucleotides between the first or second genome and the SINE consensus.

The four genomes (lag, dva, pra, and pmu (PodMur_1.0)) were compared by combining information about the pairwise comparison of the loci and were divided into categories of either “plus” for every species or one “minus” in one of the taxa.

## 3. Results

### 3.1. Detection of Squam1 SINE in the Compared Genomes

A comprehensive search for copies of Squam1 SINE in the target genomes using the ssearch36 algorithm from the FASTA package found the number of copies shown in Table 1. For comparative analysis, only copies located no closer than 300 bp from other Squam1 copies were used. Two genomic assemblies of the same species (*P. muralis*), sequenced and assembled using different methods, were compared for the total number of Squam1 copies to assess the stability of the data within a single species.

### 3.2. Search and Comparison of Orthologous Loci

**Figure 2:**
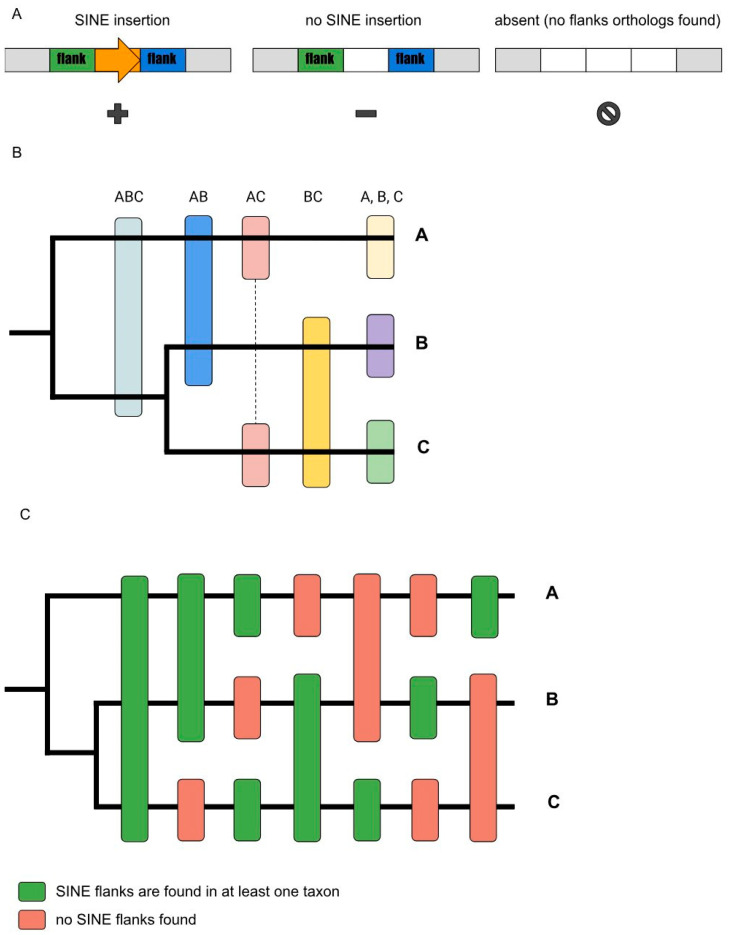
Structural variants of SINE-containing loci. (**A**) Empty blocks represent either the absence of a sequence or the absence of significant similarity to the expected sequence at the locus. (**B**) Different variants of the distribution of SINE insertions in triple-genome comparison. A, B, and C are the compared genomes. (**C**) Theoretical possibilities for the distribution of SINE loci in three species based on flanking sequences.

For each pair of species, the maximum possible number of loci in which at least one of the genomes contained a copy of Squam1 (Figure 2A,B) was found and tested for reliability.

**Figure 3:**
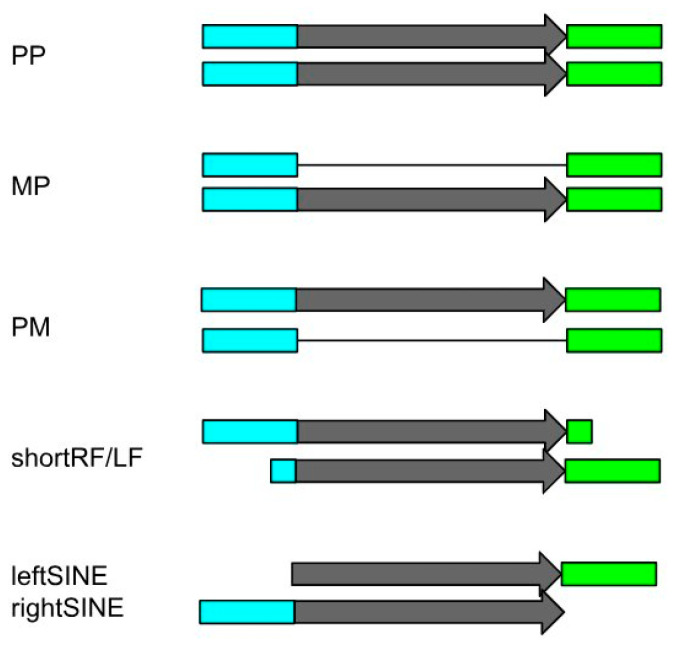
Scheme of pairwise aligned orthologous SINE loci. The three upper variants—plus–plus (PP), minus–plus (MP), and plus–minus (PM)—were considered to be reliably identified. shortRF/LF—SINE copies with flanking sequences that were too short to be reliably compared; leftSINE, rightSINE—absence of a flank in a locus due to assembly/sequencing error or difference in sequence. Loci with shortRF/LF or leftSINE/rightSINE were excluded from further analysis.

**Figure 4:**
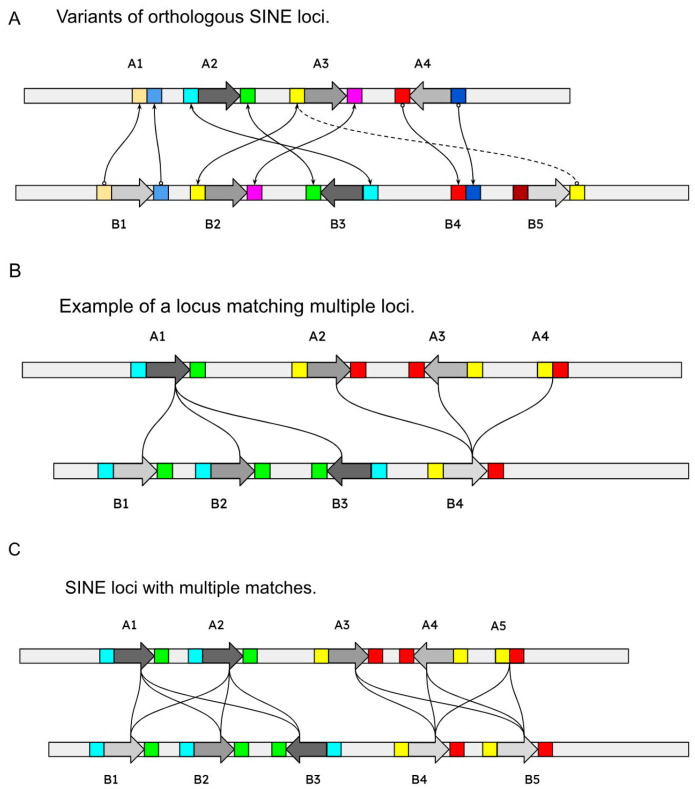
Variants of mapping flanks on the target genome. SINEs are shown as gray arrow boxes; flanks—colored boxes. A1–A5 and B1–B5—SINE loci in two genomes. (**A**) Various sets of SINE loci with matching flanks. (**B**) Multiple matching A1 loci to two locations in a target second genome. (**C**) Multiple matches both within and across the genomes.

Some copies did not find a matching pair due to differences in flanking sequences (Figure 2C and Figure 3), while others were among reliably determined pairs, and still others were part of clusters of several copies with similar flanks (Figure 4). Depending on whether the considered copy was polymorphic or not, we divided the findings into three categories: “plus-minus”, “minus-plus”, and “plus-plus”.

Pairs that did not pass the quality control (see Methods section) were manually reviewed and their unsuitability was confirmed. In the case where a locus corresponded to several similar loci in the target or its own genome, such “multicopies” were considered separately (Figure 4).

The copies in which SINE was present in both genomes (non-polymorphic copies) allow us to estimate the degree of intergenomic divergence both between the SINEs themselves and between the flanks. Polymorphic loci can also be evaluated, in addition to the criterion of the presence/absence of SINE, by the similarity of this SINE to the general consensus, as well as whether it differs from those that are found in non-polymorphic copies. Combined data for all pairs of species are represented by either shared or polymorphic insertions (Table 2).

### 3.3. Multicopies

A significant portion of the SINE loci were found to be ambiguous as their flanking sequences matched to more than one location in a target genome (“multicopies”). Manual inspection of multiple alignments of these sequences indicated that, in some of them, unrelated sequences can be filtered out on the basis of the formal parameters of the alignment. All multicopies were additionally subjected to the identification of the most similar pair, after which the locus type (polymorphic or non-polymorphic) was established. If there were no other similar loci in this cluster, such copies were considered ordinary doubles, and if there were, then they were included in the group of multilocus copies and they were not analyzed further.

Multilocus copies, in which the left flank was similar to more than three loci in two genomes, were additionally checked for the similarity of the right flanks. If the similarity was confirmed, then the flanks themselves were compared for the presence of dispersed repeats, making it difficult to establish homology. Difficulties were presented by cases when it was not possible to identify a certain general sequence motif among the members of the cluster. The total number of multilocus copies that passed a quality check was approximately 5–10%, depending on the species pair.

### 3.4. Interspecific Comparisons

Table 3 shows the number of loci for which the similarity of flanks was established among all three genomes from different genera. They were also analyzed according to the “plus-minus” scheme. As a result, both homomorphic (SINE insertion in all three species) and loci with SINE insertion in two species loci were identified, in which the absence of the insertion was confirmed in the third species.

**Figure 5:**
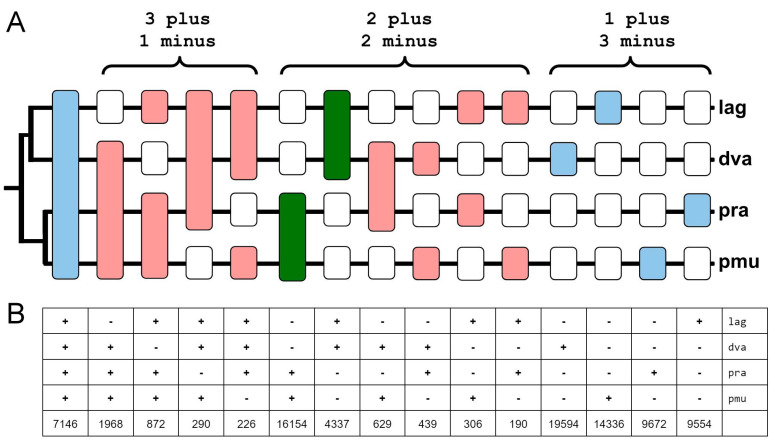
Comparison of four lizard genomes for the presence of Squam1 insertions in orthologous loci. (**A**) The possible variants of orthologous loci are shown on the hypothetical phylogenetic tree: blue—there is an insertion and the locus is phylogenetically non-informative (unique or present in all genomes); green—phylogenetically informative insertion; red—insertion contradicting the phylogenetic hypothesis; white—no insertion. (**B**) The corresponding quantitative data for each variant of interspecific comparison.

A more detailed study using the example of four species, including two closely related *Podarcis* species, allowed us to observe the degree of polymorphism of the loci against the background of the level of interspecific differences between species of the same genus (Figure 5).

## 4. Discussion

### 4.1. Possible Variants of Orthologous Loci Containing Interspersed Repeats

The intergenomic comparison of the flanking sequences of SINE copies allows us to establish their pairwise and multiple matches. Depending on the parameters of the minimum required similarity, the flank may either not find a reliable match in the target genome or find one such match, or there may be several such matches. In the first case, the probability of finding an ortholog decreases to zero, since if such an ortholog still exists, to detect it, one would have to change the selection criteria, either by lowering the similarity threshold or by increasing the length of the flanks. A genome locus of this type can have too many single mutations or undergo a large indel event that makes the flank unrecognizable. The question of whether such a copy can be considered a derived feature lies in the plane of confidence in the quality of the sequencing of the analyzed genomic region, and may require additional verification, for example, using PCR (although the absence of the product in the target species does not strictly mean the absence of an ortholog) or a detailed analysis of the sequences surrounding the SINE sequence (whether they contain, for example, the insertion of another mobile element). In the case of unambiguous similarity, the pair of sequences has the characteristics of an ortholog, and such copies carry the most reliable information about the possible polymorphism of SINE insertions. In more stringent requirements, it may additionally be necessary to verify the non-randomness of such similarity, either by increasing the flanks or by applying data from the whole-genome alignment, if available, based on chromosomal location or known genomic environment (for example, near certain genes). Dividing loci into “plus-plus”, “plus-minus”, and “minus-plus” categories allows these data to be used to estimate the degree of similarity of genomes in general terms, as well as the differentiated rates of divergence after diversification based on the number of specific insertions.

The greatest difficulties are presented by the analysis of multiple matches. One locus in the original genome can correspond to several hits in the target genome, some of which, in turn, find correspondence to several more loci in the original genome, which in total create a set of mutually linked loci. Typically, in works on SINE polymorphism, such loci are excluded from consideration. In our opinion, consideration of such cases will make it possible in the future not only to increase the number of available characters for intergenomic comparison, but also to identify structural and/or biological features of such copies associated with the phenomenon of multimapping. The reasons leading to the similarity of flanking sequences may be different in form and nature, and may or may not be related to the peculiarities of emergence and subsequent evolution of SINEs themselves, adjacent other mobile elements or other genomic sequences. Therefore, we hope that an effective approach to their identification and characterization will lead to a more complete understanding of aspects of SINE evolution, including in non-model organisms. We addressed this problem from several positions.

First, a cluster of linked loci is checked to see if there is a most similar pair from different species among them, which has a reliable level of similarity, compared to which all other members of the cluster have much less similarity. In this case, it is possible to leave only this pair, excluding the remaining loci from consideration.

Second, if such a cluster has more than two elements with comparable levels of similarity, then attention should be paid to the specific sequence that forms the flank in order to try to identify its origin (for example, a mobile element, microsatellite, etc.). If it is assumed that the recent insertion of a mobile element or other event has modified the flank, then a reasonable increase in the length of the flanks and a new consideration of their similarity can shed light on the establishment of reliable similarity between the elements of the cluster. In any case, such clusters should be considered separately from pairwise loci from the previous category due to the features of their structure and, probably, the mutations that occurred in their flanks.

### 4.2. The Similarity in Pairwise Comparisons Reflects the Phylogenetic Distance between Taxa

The results of the pairwise analysis of Squam1 loci in the four lizard species (Table 2) can be considered in the context of the presumed relationship of the taxa, although the exact scheme of the relationships of the three genera used in our work has not been firmly established. There is a comparable level of common “plus-plus” loci in pairs of species from different genera, while an increased number of common loci was found in *Podarcis* genomes. This may be explained, on the one hand, by the constancy of the SINE loci themselves due to the relatedness of the species, as well as by the greater similarity of the flanks and, as a consequence, the more successful identification of orthologous loci. In different species pairs, a complex picture is observed, both in terms of the ratio between constant (“plus-plus”) and unique (“plus-minus” or “minus-plus”) loci, as well as the absolute number of unique loci, which can reflect the degree of SINE activity after divergence in this species pair, as well as mutational changes occurring in the surrounding loci (nearest flanks and more distant sequences). Such processes can make it difficult or even impossible to establish the orthology. It is necessary to estimate the contribution of two different processes (the own evolutionary dynamics of SINE against the background of flanking divergence), using various quantitative parameters of the loci determined in the proposed analysis of orthologous alignments. With more detailed consideration, it seems possible to estimate the temporal dynamics of the formation of new copies in those taxa for which the order and times of divergence are well known.

### 4.3. Quadruple Alignment Analysis

When analyzing four genomes, the similarity of the two *Podarcis* species is clearly evident against the background of the divergence of representatives of different genera (Figure 5). The number of loci shared by all four, as well as the number of phylogenetically consistent loci in each category (according to the number of plus–minus loci, indicated at the top of Figure 5), significantly exceeds the number that do not fit into the proposed scheme of taxon divergence. The evolution of SINE-containing loci, indicated by our quantitative data, reflects the order of divergence and can potentially serve as the basis for a more detailed analysis of the rates of accumulation of differences at different stages of taxon diversification. A more detailed examination of alignments of individual loci shows that in cases contradicting phylogeny, mutations decreasing a similarity due to random variations are more numerous. However, a formal analysis of such parameters is difficult due to the extreme diversity of such mutations. In general, the data indicate intense mutational processes affecting SINE loci, both at the level of intergeneric comparisons and between species of the same genus. The appearance of unique SINE insertions in different species, although it does not carry information about the branching order, indicates the rate of evolution after the diversification of terminal branches, and differs significantly in, for example, the sand lizard and *Podarcis*—more than twice. Apparently, the processes associated with changes in SINE loci occur due to both their own retropositional activity and the mutability of the insertion regions. This allows us to find reliable (orthology-related) differences at different levels of distance between taxa, which can be useful when comparing species and genera, the relationships of which are difficult to establish using other types of data.

### 4.4. Methodological Difficulties and Possible Ways to Overcome Them

The productivity of each stage of the identification and analysis of SINE loci directly depends on the choice of method and the parameters embedded in it. In this section, we will discuss the challenges of the pairwise analysis of SINE loci.

At the first stage—the search for individual copies of the selected SINE family in a full-genome assembly—it is important to choose a suitable SINE consensus sequence and a filtering threshold for the found copies, since many subfamilies of different structures and sequences can be present in the genome. Frequently occurring truncated and highly diverged copies, especially in old SINE families and between distant taxa, also lead to the need to carefully weigh these parameters when searching for repeats in each genome.

Mapping of the flanking sequences, in our case using the BWA-MEM program, should also be adjusted for a specific object. The length of the flanks should be, on the one hand, sufficient to allow for their unambiguous identification; on the other hand, they should not lead to false negative results in those loci where there is a high level of diversification of sequences. Filtering the mapping results by length can remove many false-positive hits, but simply rejecting results with a low mapping quality parameter, as well as possible multimapping ones, according to our selective manual inspection of particular loci, is unjustified. It is more correct to perform additional filtering at a later stage of sequence alignment.

The most difficult interpretation is the results of multimappers—cases where at least one flank of a SINE copy corresponds to several hits in the target genome. Simply discarding such cases, in our observations, significantly depletes the results. We propose a strategy of dividing these cases into two categories: multimappers with many repeats (more than 10 hits) and multimappers with a moderate number of repeats (up to 10 hits). In the case of the first, the routine analysis of such cases runs into insurmountable difficulties and must be carried out taking into account which specific multicopy sequences lead to numerous detections of similarity in the flanks. Possible approaches are filtering of false-positive hits through a numerical assessment of the similarity of the flanks, as well as, if possible, selecting different flanking sequences, located at some distance from the site of the SINE insertion. However, in this case, it is hardly possible to unambiguously determine whether it is justified to compare such multimappers with the remaining non-multimapper loci. Other methods that have been successfully used to identify SINE insertions between genomes can help to clarify the genomic context and the presence/absence status of such problematic loci. For example, the 2-n-way method, which is based on the whole-genome alignments, offers the possibility of obtaining such information and subsequently analyzing the extended genomic context of the candidate loci [12].

In the case of a not-so-large number of multimappers, a strategy was found to be effective, in which a pair of the most similar sequences from different genomes is selected from the multiple alignment of the candidate loci. Such a pair is analyzed for reliability and for the presence or absence of SINE in the loci. After that, in case of a positive result, those from the original set of loci are selected in which the similarity with this pair exceeds a certain threshold.

The obtained set of paired loci in our approach goes through a step-by-step check for reliability and type. Initially, loci are discarded where the SINE is too close to the beginning of the locus and the flanks are too short. Next, the length of the similarity and the number of identical nucleotides is determined for the flanks. At the final stage, the SINE is compared with the consensus and between the loci, and the degree of similarity of the SINE to each other and to the consensus is determined.

Obtaining such parameters allows for fine-tuning the identification of loci in accordance with the characteristics of SINE. For example, special attention can be paid to indels, if SINE tends to accumulate them in its nucleotide sequence, or the percentage of similarity, if the degree of its divergence is important, for example, when searching for the youngest, most recently integrated copies. It was often the case that where the SINE was located in one of the genomes compared, there was an unrelated sequence of similar length in the other genome. Such cases can either be considered variants with no SINE insertion or be filtered out due to non-compliance with the parameters. The “foreign” sequences themselves in these cases can be extracted and analyzed to clarify their origin. The intermediate alignments obtained at each stage allow for easy visual tracking of the results of the alignments, and for the reconfiguration of the parameters, which can significantly affect the final result.

### 4.5. Applications of the Described Approaches and Information That Can Be Obtained from the Alignments

The collected statistics allow us to establish the following properties of loci. Numerical data obtained from orthologous loci can be binary (presence/absence of a copy) and reflect the degree of divergence of sequences and the affiliation of polymorphic or homomorphic orthologous loci to certain subfamilies of SINE. The genomic location of copies, in particular, relative to genes and other known elements of the genome, can also be important. In cases where, in the absence of SINE at a locus, there is a sequence unrelated to SINE, the frequency of their occurrence, the degree of interspecific differences, and the origin of such sequences are of interest for understanding genome evolution.

It appears promising to compare multiple different SINE families, as they can evolve in different ways and reflect genome divergence from different angles. In this case, it becomes possible to select the SINE family or subfamily whose rates of accumulation of new copies or mutation of old copies correspond to the divergence of the genomes of compared taxa.

In general, the approach proposed allows us to obtain numerous data on the comparison of SINE-containing orthologous loci in several hours, which provides a wealth of material for the comparative quantitative and qualitative analysis of the similarity and differences in the compared genomes and their parts, as well as of the dispersed DNA repeats themselves.

## Figures and Tables

**Table 1:** Number of Squam1 copies found.

	*L. agilis*	*D. valentini*	*P. raffonei*	*P. muralis1*	*P. muralis2*
Squam1 total	65,094	99,365	107,217	130,894	129,109
Squam1 distant	62,951	94,910	101,377	121,860	120,373

Squam1 total—total number of Squam1 SINE copies; Squam1 distant—copies located more than 300 bp apart. Sources for *Podarcis muralis* genome assemblies are *P. muralis1*—LU_Pmuni_1.1 and *P. muralis2*—PodMur_1.0.

**Table 2:** Number of loci found in pairwise comparison of lizard genomes.

	pmu-pra	dva-pmu	lag-pmu	dva-pra	lag-pra	dva-lag
MP	19,914	43,508	46,662	38,205	41,619	16,725
PM	31,580	34,354	20,449	36,474	21,707	35,257
PP	50,016	12,896	12,098	13,644	12,549	17,831

MP—minus locus in first species of a pair and plus in a second species; PM—plus locus in first genome and minus locus in second genome; PP—plus insertion in both genomes; lag—*Lacerta agilis*; dva—*Darevskia valentini*; pra—*Podarcis raffonei*; pmu—*Podarcis muralis*.

**Table 3:** The number of loci with Squam1 insertions in all three species (LDP) or only in two (LD, LP, PD).

LDP	LD	LP	PD
7388	4637	967	3437

L—*Lacerta agilis*; D—*Darevskia valentini*; P—*Podarcis raffonei*.

## Data Availability

The script used for data processing is available at: https://github.com/Toki-bio/SINE_orth_loc/, accessed on 1 October 2023.

## Supplementary Materials

The following supporting information can be downloaded at: https://www.mdpi.com/article/10.3390/genes14112089/s1.
