# Supplementary material for: Interspecific Comparison of Orthologous Short Interspersed Elements Loci Using Whole-Genome Data
Source: Genes (Basel). 2023 Nov 17;14(11):2089. doi: 10.3390/genes14112089 (PMC10670947; doi:10.3390/genes14112089)
Supplement: Supplementary file 1 [file genes-14-02089-s001.zip › Supplemental File 3.pdf]

Supplementary material for Kosushkin et al, 2023.

A workflow exemplified by the comparison of Squam1 SINE-containing loci in *Lacerta agilis* (**lag**) and *Darevskia valentini* (**dva**) genome assemblies (files named lag.bnk and dva.bnk with corresponding .fai indices).

First, all copies of Squam1 are found in both genomes and the results are collected in .bed files (here named lag-Squam1.bed and dva-Squam1.bed).

For each step, a brief description is given, for some steps together with a short sample of the input and output data.

Input files are shown in red, output files are in blue.

Several lines of input and output data are provided for some steps.

When the term “locus” is used, it means “a locus in either genome that contains a SINE insertion in at least one of the genomes”.

A consensus sequence of the Squam1 SINE used in this work should be called squam1.q:

```
>squam1
gggacccaggtggcgctgtgggtTAaAcCAcagagCCTaGGGCTTGCcGATCAGAAGGTCgGcgGTTCgAATCCCtGcgaCGGGGTGAGCTCCC
GTTGCTCGGTCCCtGCTCCTGCCaACCTAGCAGTTCgAAAGCACgtCAAAGTGCAAGTAGATAAATAGGTACCgCTccggcGGGAAG
GTAAACgGCGTTTCcGTGCgCTGCTCTGGTTcGCCAGAAGCgGCTTaGTCATGCTGgCCACATGACCnGGAAgCTGTAcgCcgGCTC
CCTCgGCCaaTAAaGcGAGATGAGCGCCgCAACCCCAGAGTcGgcacgAcTGgACcTAAtgGTcaGGGGTcCCTTTACCTTtacctttt
```

This particular sequence was obtained by multiple sequence alignment of randomly extracted copies from the **dva** genome.

Please note that this text is not intended to be a stand-alone script but only to illustrate how the code works; actual working version of a script can be found on github https://github.com/Toki-bio/SINE_orth_loc

1. Flanks preparation and mapping

1.1. Select SINE copies that are more than 300bp apart.

```
sort -k1,1 -k2,2n lag-Squam1.bed | bedtools cluster -d 300 -i - > lag-Squam1_clust.bed

uniq -f6 -u lag-Squam1_clust.bed > lag-Squam1_uniq.bed # SINE loci separated by more than 300bp

uniq -f6 -D lag-Squam1_clust.bed > lag-Squam1_clust.bed_clusters.bed # SINE loci that are within 300 bp from each other (excluded from downstream analysis)
```

```
sort -k1,1 -k2,2n dva-Squam1.bed | bedtools cluster -d 300 -i - > dva-Squam1_clust.bed

uniq -f6 -u dva-Squam1_clust.bed > dva-Squam1_uniq.bed

uniq -f6 -D dva-Squam1_clust.bed > dva-Squam1_clusters.bed
```

1.2. Prepare 300bp flanks for each SINE locus from previous step.

```
bedtools flank -s -i lag-Squam1_uniq.bed -g lag.fai -l 300 -r 0 > lag_flanks_300.bed # creating 300bp left flanks for all SINE loci

bedtools getfasta -s -bed lag_flanks_300.bed -fi lag.bnk > lag_flanks_300.bnk

bedtools flank -s -i dva-Squam1_uniq.bed -g dva.fai -l 300 -r 0 > dva_flanks_300.bed

bedtools getfasta -s -bed dva_flanks_300.bed -fi dva.bnk > dva_flanks_300.bnk
```

1.3. Mapping of flanking sequences from source genomes to target genomes

```
bwa mem -t=$(nproc) lag.bnk dva_flanks_300.bnk > dva_flanks-lag.sam

sam2bed < dva_flanks-lag.sam | awk '{if (length($12)>99) print $1,$2,$3,$4,"MAPQ="$5",LENGTH="length($12)","$16,$6}' |

awk '{sub(/:i:/,"=",$5)}1' OFS="\t" | bedtools sort -i > dva_flanks-lag.bed


bwa mem -t=$(nproc) dva.bnk lag_flanks_300.bnk > lag_flanks-dva.sam

sam2bed < lag_flanks-dva.sam | awk '{if (length($12)>99) print $1,$2,$3,$4,"MAPQ="$5",LENGTH="length($12)","$16,$6}' |

awk '{sub(/:i:/,"=",$5)}1' OFS="\t" | bedtools sort -i > lag_flanks-dva.bed
```

As a result of this step, two bed files are created, each containing coordinates for each match to a left (5') SINE flank sequence (columns 1, 2, and 3 in the bed file) with a corresponding coordinate of a putative matching locus in the target genome (column 4 in the bed file).

Working with **lag** coordinates mapped on the **dva** genome

1.4. Genomic coordinates for each flank in each species increased by 600bp downstream from the left flank. The resulting interval is expected to cover the left flank, SINE (in case there is an inserted copy at a given locus) and right (5') flank.

```
bedtools slop -s -i lag_flanks-dva.bed -g dva.bnk.fai -l 0 -r 600 > lag_flanks-dva_slop600.bed
```

sample of input data:

```
dva1  45334  45631  lag1:74097241-74097541(-)  MAPQ=60,LENGTH=300,AS=240  +
dva1  47966  48251  lag1:74093567-74093867(+)  MAPQ=60,LENGTH=300,AS=232  -
dva1  74452  74749  lag1:74067116-74067416(+)  MAPQ=60,LENGTH=300,AS=247  -
```

sample of output data:

```
dva1  45334  46288  lag1:74097241-74097541(-)  MAPQ=60,LENGTH=300,AS=240  +
dva1  47309  48251  lag1:74093567-74093867(+)  MAPQ=60,LENGTH=300,AS=232  -
dva1  73795  74749  lag1:74067116-74067416(+)  MAPQ=60,LENGTH=300,AS=247  -
```

1.5. Swap the data fields (coordinates from the target genome are written from column 4 to columns 1, 2, and 3) and slop dva coordinates

```
awk -F "[, ]+" 'BEGIN{OFS=FS="\t"}; {sub( /-/, "\t", $4); sub( /:/, "\t", $4); { gsub(/\)/, "")}; { gsub(/\(/, "\t0\t0\t")}; {print $4, $5, $6, $1":"$2"-"$3"("$11")", $10, $9}; }' lag_flanks-dva_slop600.bed | bedtools sort | bedtools slop -s -i - -g lag.bnk.fai -l 0 -r 600 | awk '{print $0"\tlag_mapped_on_dva"}' > inv_lag_flanks-dva_slop600.bed
```

sample of output data:

```
lag1  3589    4546    dva11:12095069-12095992(-)    MAPQ=21,LENGTH=300,AS=171  +  lag_mapped_on_dva
lag1  72812   73769   dva121:1351687-1352651(+)     MAPQ=60,LENGTH=300,AS=232  -  lag_mapped_on_dva
lag1  142003  142960  dva121:1270396-1271352(-)     MAPQ=60,LENGTH=300,AS=213  +  lag_mapped_on_dva
```

Working with **dva** coordinates mapped on **lag** genome

1.6. Same as in the previous steps (1.4 and 1.5) but for a different mapping direction: slop coordinates for **dva** by 600bp

```
bedtools slop -s -i dva_flanks-lag.bed -g lag.bnk.fai -l 0 -r 600 > dva_flanks-lag_slop600.bed
```

sample of input data:

```
lag1  21535   21748   dva121:1402761-1403061(+)  MAPQ=60,LENGTH=300,AS=152  -
lag1  73469   73762   dva121:1351694-1351994(+)  MAPQ=60,LENGTH=300,AS=225  -
lag1  142002  142303  dva121:1271053-1271353(-)  MAPQ=13,LENGTH=300,AS=214  +
```

sample of output data:

```
lag1  20878   21748   dva121:1402761-1403061(+)  MAPQ=60,LENGTH=300,AS=152  -
lag1  72812   73762   dva121:1351694-1351994(+)  MAPQ=60,LENGTH=300,AS=225  -
lag1  142002  142960  dva121:1271053-1271353(-)  MAPQ=13,LENGTH=300,AS=214  +
```

1.7. Invert the data fields and slop **lag** coordinates

```
awk -F "[, ]+" 'BEGIN{OFS=FS="\t"}; {sub( /-/, "\t", $4); sub( /:/, "\t", $4); { gsub(/\)/, "")}; { gsub(/\(/, "\t0\t0\t")}; {print $4, $5, $6, $1":"$2"-"$3"("$11")", $10, $9}; }' dva_flanks-lag_slop600.bed | bedtools sort | bedtools slop -s -i - -g dva.bnk.fai -l 0 -r 600 | awk -F "[, ]+" 'BEGIN{OFS=FS="\t"}; {sub( /-/, "\t", $4); sub( /:/, "\t", $4); { gsub(/\)/, "")}; { gsub(/\(/, "\t0\t0\t")}; {print $4, $5, $6, $1":"$2"-"$3"("$11")", $10, $9}; }' | bedtools sort | awk '{print $0"\tdva_mapped_on_lag"}'> dva_flanks-lag_slop600_2i.bed
```

sample of output data:

lag1	20878	21748	dva121:1402761-1403718(+)	MAPQ=60,LENGTH=300,AS=152	-	dva_mapped_on_lag
lag1	72812	73762	dva121:1351694-1352651(+)	MAPQ=60,LENGTH=300,AS=225	-	dva_mapped_on_lag
lag1	142002	142960	dva121:1270396-1271353(-)	MAPQ=13,LENGTH=300,AS=214	+	dva_mapped_on_lag

The result is four datasets are produced (two for each species) with mapped left flank coordinates slopped by 600bp.

1.8. Merge datasets paired in two .bed files into a combined dataset..

```
cat dva_flanks-lag_slop600_2i.bed inv_lag_flanks-dva_slop600.bed | sort -k1,1 -k2,2n > lag-dva_m.bed
```

sample of output data:

lag1	3589	4546	dva11:12095069-12095992(-)	MAPQ=21,LENGTH=300,AS=171	+	lag_mapped_on_dva
lag1	20878	21748	dva121:1402761-1403718(+)	MAPQ=60,LENGTH=300,AS=152	-	dva_mapped_on_lag
lag1	72812	73762	dva121:1351694-1352651(+)	MAPQ=60,LENGTH=300,AS=225	-	dva_mapped_on_lag

Each row contains the full coordinates (left flank+SINE+right flank) of the **lag** locus against corresponding **dra** locus, and vice versa. The direction of mapping is given in the last column. Three important mapping parameters are retained in the 5th column: mapping quality (MAPQ), length of the left flank alignment, and alignment score generated by the aligner (AS) as they are provided in the .sam file.

2. Clustering

Overlapping sets of coordinates are combined into clusters which may contain pairs or multiple corresponding sets of loci.

In the case of a one-to-one match between genomes, two lines of data are expected for a cluster. Multiple matches from the mapping results are represented by more than two lines.

2.1. Clustering of overlapping lag loci: **lag** loci are clustered based on the overlap of their coordinates; the corresponding **dra** coordinates are given in column 4.

```
bedtools cluster -s -i lag-dva_m.bed | awk 'OFS="\t" {print $1,$2,$3,$4,$5,$6,"lag"$8"c",$7}'> clu_lag.bed
```

sample of output data:

lag1	3589	4546	dva11:12095069-12095992(-)	MAPQ=21,LENGTH=300,AS=171	+	lag1c	lag_mapped_on_dva
lag1	142002	142960	dva121:1270396-1271353(-)	MAPQ=13,LENGTH=300,AS=214	+	lag2c	dva_mapped_on_lag
lag1	142003	142960	dva121:1270396-1271352(-)	MAPQ=60,LENGTH=300,AS=213	+	lag2c	lag_mapped_on_dva

In column 7, names of lag clusters are given.

2.2. Move **lag** loci coordinates to column 4; **dra** coordinates are written in columns 1, 2, and 3 and then clustered.

```
awk -F "[, ]+" 'BEGIN{OFS=FS="\t"}; {sub( /-/, "\t", $4); sub( /:/, "\t", $4); { gsub(/\)/, "")}; { gsub(/\(/, "\t0\t0\t")}; {print $4, $5, $6, $1":"$2"-"$3"("$11")", $10, $9, $12}; }' lag-dva_m.bed | bedtools sort | bedtools cluster -s -i - | awk 'OFS="\t" {print $1,$2,$3,$4,$5,$6,"dva"$8"c",$7}'> clu_dva.bed
```

sample of output data:

dva1	29592	30549	lag1:74112621-74113562(-)	MAPQ=60,LENGTH=300,AS=199	+	dva1c	dva_mapped_on_lag
dva1	45333	46290	lag1:74096586-74097542(-)	MAPQ=60,LENGTH=300,AS=241	+	dva2c	dva_mapped_on_lag
dva1	45334	46288	lag1:74096584-74097541(-)	MAPQ=60,LENGTH=300,AS=240	+	dva2c	lag_mapped_on_dva

2.3 Cluster **dva** loci obtained from swapped result of step 2.1.

```
awk -F "[, ]+" 'BEGIN{OFS=FS="\t"}; {sub( /-/, "\t", $4); sub( /:/, "\t", $4); { gsub(/\)/, "")}; { gsub(/\(/, "\t0\t0\t")}; {print $4, $5, $6, $1":"$2"-"$3"("$11")", $10, $9, $12, $13}; }' clu_lag.bed > dva_from_clu_lag.bed
```

sample of output data:

dva11	12095069	12095992	lag1:3589-4546(+)	MAPQ=21,LENGTH=300,AS=171	-	lag1c	lag_mapped_on_dva
dva121	1270396	1271353	lag1:142002-142960(+)	MAPQ=13,LENGTH=300,AS=214	-	lag2c	dva_mapped_on_lag
dva121	1270396	1271352	lag1:142003-142960(+)	MAPQ=60,LENGTH=300,AS=213	-	lag2c	lag_mapped_on_dva

2.4. Cluster **lag** loci obtained from swapped result of step 2.2.

```
awk -F "[, ]+" 'BEGIN{OFS=FS="\t"}; {sub( /-/, "\t", $4); sub( /:/, "\t", $4); { gsub(/\)/, "")}; { gsub(/\(/, "\t0\t0\t")}; {print $4, $5, $6, $1":"$2"-"$3"("$11")", $10, $9, $12, $13}; }' clu_dva.bed > lag_from_clu_dva.bed
```

sample of output data:

lag1	74112621	74113562	dva1:29592-30549(+)	MAPQ=60,LENGTH=300,AS=199	-	dva1c	dva_mapped_on_lag
lag1	74096586	74097542	dva1:45333-46290(+)	MAPQ=60,LENGTH=300,AS=241	-	dva2c	dva_mapped_on_lag
lag1	74096584	74097541	dva1:45334-46288(+)	MAPQ=60,LENGTH=300,AS=240	-	dva2c	lag_mapped_on_dva

The output of steps 2.1-2.4 are four data sets (two for each genome) of coordinates of loci, for each of which SINE is present in at least one genome, with cluster names in column 5.

2.5. Merge all data sets from each genome

```
cat lag_from_clu_dva.bed clu_lag.bed | sort -k1,1 -k2,2n > lag_comb_clu.bed
```

sample of output data:

lag1	3589	4546	dva11:12095069-12095992(-)	MAPQ=21,LENGTH=300,AS=171	+	dva7139c	lag_mapped_on_dva
lag1	3589	4546	dva11:12095069-12095992(-)	MAPQ=21,LENGTH=300,AS=171	+	lag1c	lag_mapped_on_dva
lag1	20878	21748	dva121:1402761-1403718(+)	MAPQ=60,LENGTH=300,AS=152	-	dva11071c	dva_mapped_on_lag

```
cat dva_from_clu_lag.bed clu_dva.bed | sort -k1,1 -k2,2n > dva_comb_clu.bed
```

sample of output data:

dva1 29592 30549 lag1:74112621-74113562(-) MAPQ=60,LENGTH=300,AS=199 + dva1c dva_mapped_on_lag
dva1 29592 30549 lag1:74112621-74113562(-) MAPQ=60,LENGTH=300,AS=199 + lag8699c dva_mapped_on_lag
dva1 45333 46290 lag1:74096586-74097542(-) MAPQ=60,LENGTH=300,AS=241 + dva2c dva_mapped_on_lag

2.6. Merge the two data sets for later analysis

```
cat lag_comb_clu.bed dva_comb_clu.bed > lagdva
```

sample of output data:

dva10004 760 1575 C57866R MAPQ=13,LENGTH=300,AS=92 + lag2:112701673-112702630(+)
lag_mapped_on_dva
dva10007 1261 2218 C57875R MAPQ=0,LENGTH=300,AS=40 - lag5:277209-277926(-) dva_mapped_on_lag
dva10013 1832 2789 C57893R MAPQ=60,LENGTH=300,AS=172 - lag20:37296913-37297846(+)
dva_mapped_on_lag

2.7. Merge all **lag** loci while retaining cluster names

```
bedtools merge -i lag_comb_clu.bed -c 4,5,6,7,8 -o collapse,collapse,collapse,collapse,collapse > lag_comb_clu_mer.bed
```

sample of output data:

lag1 3589 4546 dva11:12095069-12095992(-),dva11:12095069-12095992(-)
MAPQ=21,LENGTH=300,AS=171,MAPQ=21,LENGTH=300,AS=171 +,+ dva7139c,lag1c lag_map
ped_on_dva,lag_mapped_on_dva
lag1 20878 21748 dva121:1402761-1403718(+),dva121:1402761-1403718(+)
MAPQ=60,LENGTH=300,AS=152,MAPQ=60,LENGTH=300,AS=152 -,- dva11071c,lag5649c
dva_mapped_on_lag,dva_mapped_on_lag
lag1 72812 73769
dva121:1351694-1352651(+),dva121:1351694-1352651(+),dva121:1351687-1352651(+),dva121:1351687-1352651(+)
MAPQ=60,LENGTH=300,AS=225,MAPQ=60,LENGT
H=300,AS=225,MAPQ=60,LENGTH=300,AS=232,MAPQ=60,LENGTH=300,AS=232 -,-,-,- dva11070c,lag5650c,dva11070c,lag5650c
dva_mapped_on_lag,dva_mapped_on_lag,lag_mapped_on_dva,
lag_mapped_on_dva

2.8. Merge all **dva** loci while retaining cluster names

```
bedtools merge -i dva_comb_clu.bed -c 4,5,6,7,8 -o collapse,collapse,collapse,collapse,collapse > dva_comb_clu_mer.bed
```

sample of output data:

dva1 29592 30549 lag1:74112621-74113562(-),lag1:74112621-74113562(-)
MAPQ=60,LENGTH=300,AS=199,MAPQ=60,LENGTH=300,AS=199 +,+ dva1c,lag8699c dva_map
ped_on_lag,dva_mapped_on_lag
dva1 45333 46290
lag1:74096586-74097542(-),lag1:74096586-74097542(-),lag1:74096584-74097541(-),lag1:74096584-74097541(-)
MAPQ=60,LENGTH=300,AS=241,MAPQ=60,LENGT
H=300,AS=241,MAPQ=60,LENGTH=300,AS=240,MAPQ=60,LENGTH=300,AS=240 +,+,+,+ dva2c,lag8698c,dva2c,lag8698c
dva_mapped_on_lag,dva_mapped_on_lag,lag_mapped_on_dva,lag_mapp
ed_on_dva
dva1 47309 48251 lag1:74093567-74094524(+),lag1:74093567-74094524(+)
MAPQ=60,LENGTH=300,AS=232,MAPQ=60,LENGTH=300,AS=232 -,- dva1454c,lag3083c
lag_mapped_on_dva,lag_mapped_on_dva

The output files contain cluster names for each locus in column 7.

3. Working with clusters

3.1. Combining clusters for both genomes, keeping only cluster names

```
awk '{print $7}' dva_comb_clu_mer.bed lag_comb_clu_mer.bed > lag-dva_clusters
awk '{gsub(/,/,"\t")}1' lag-dva_clusters > lag-dva_clust.ters
```

sample of output data:

```
dva1c,lag8699c
dva2c,lag8698c,dva2c,lag8698c
dva1454c,lag3083c
```

3.2. Remove duplicated values in each line of data

```
awk '{ delete seen ; for(i=1;i<=NF;i++) if ( ! ($i in seen) ) { printf "%s ",$i ; seen[$i]=i ; } ; printf "\n"}' lag-dva_clust.ters | awk '!seen[$0]++' > lag-dva_clust.uniq
```

3.3. Combine all mutually connected clusters into "superclusters"

```
awk '{
    for ( fldNrA=1; fldNrA<NF; fldNrA++ ) {
        fldvalA = $fldNrA
        for ( fldNrB=fldNrA+1; fldNrB<=NF; fldNrB++ ) {
            fldvalB = $fldNrB
            val_pairs[fldvalA][fldvalB]
            val_pairs[fldvalB][fldvalA]
        }
    }
}

function descend(fldvalA,       fldvalB) {
    if ( !seen[fldvalA]++ ) {
        all_vals[fldvalA]
        for ( fldvalB in val_pairs[fldvalA] ) {
            descend(fldvalB)
        }
    }
}

END {
    PROCINFO["sorted_in"] = "@ind_str_asc"
    for ( fldvalA in val_pairs ) {
        delete all_vals
```

```
        descend(fldvalA)
        if ( fldvalA in all_vals ) {
            sep = ""
            for ( fldvalB in all_vals ) {
                printf "%s%s", sep, fldvalB
                sep = OFS
            }
            print ""
        }
    }
}' lag-dva_clust.uniq > lag-dva_clust.uniq.output.clusters
```

sample of output data:

```
dva100000c lag120603c
dva100001c lag120604c
dva100002c lag120605c
```

3.4. Create a list of clusters to work with

```
awk '{print "C"NR"R",$0}' lag-dva_clust.uniq.output.clusters | awk '{i = 2}{ while ( i <= NF ) { print $1,$i ; i++ } }' > list
```

sample of output data:

```
C1R dva100000c
C1R lag120603c
C2R dva100001c
```

Each "supercluster" is given a unique name.

3.5. Each locus from a list of **lag** and **dva** loci is assigned a "supercluster" name in column 4.

```
awk 'NR==FNR{a[$2]=$1;next} $7 in a {$7=a[$7]}1' OFS="\t" list lagdva | awk '{print $1,$2,$3,$7,$5,$6,$4,$8}' OFS="\t" | sort | uniq
> lagdva.bed
```

sample of output data:

```
dva10004	760	1575	C57866R MAPQ=13,LENGTH=300,AS=92	+	lag2:112701673-112702630(+)
lag_mapped_on_dva
dva10007	1261	2218	C57875R MAPQ=0,LENGTH=300,AS=40 -	lag5:277209-277926(-)	dva_mapped_on_lag
dva10013	1832	2789	C57893R MAPQ=60,LENGTH=300,AS=172	-	lag20:37296913-37297846(+)
dva_mapped_on_lag
...
lag9	9995177 9995935 C72366R MAPQ=60,LENGTH=104,AS=73	+	dva25631:127740-128697(-)	dva_mapped_on_lag
lag9	9998992 9999949 C72365R MAPQ=60,LENGTH=300,AS=197	+	dva25631:123287-124216(-)	lag_mapped_on_dva
lag9	9999020 9999949 C72365R MAPQ=60,LENGTH=300,AS=197	+	dva25631:123287-124244(-)	dva_mapped_on_lag
```

3.6. Merge all loci while keeping the name of each "supercluster"

```
bedtools sort -i lagdva.bed | bedtools merge -s -c 4,5,6 -o distinct,collapse,distinct -i - | awk '{print $1,$2,$3,$4":"$5,"0",$6}' OFS="\t" > lagdva_m.bed
```

sample of output data:

```
dva1	29592	30549	C36121R:MAPQ=60,LENGTH=300,AS=199	0	+
dva1	45333	46290	C45864R:MAPQ=60,LENGTH=300,AS=241,MAPQ=60,LENGTH=300,AS=240	0	+
dva1	47309	48251	C30647R:MAPQ=60,LENGTH=300,AS=232	0	-
…
lag9	74721422	74722379	C7232R:MAPQ=60,LENGTH=300,AS=210	0	-
lag9	74736818	74737785	C10176R:MAPQ=7,LENGTH=300,AS=102,MAPQ=22,LENGTH=300,AS=210	0	+
lag9	74745160	74745885	C40166R:MAPQ=0,LENGTH=300,AS=48	0	-
```

These steps ensure that all possible loci connections (see Fig. 3 in the manuscript) are considered and that all groups of loci are treated according to the number of loci they contain and the properties of their alignments.

4. Data extraction

4.1. Merging banks

```
cat lag.bnk dva.bnk > lag-dva.bnk
samtools faidx lag-dva.bnk
```

For convenience, two genomes are merged for subsequent extraction of fasta sequences.

4.2. Extract fasta sequences from a combined bank of two genomes and write each cluster in a single line

```
bedtools getfasta -s -name -fi lag-dva.bnk -bed lagdva_m.bed | awk -F: '{if ($0 ~ ">") {print substr($1,2),">"$4":"$5"::"$2} else print}' | awk 'NR%2{printf "%sSeqStart",$0;next;}1' | awk '{  if ($1 in clusters) clusters[$1] = clusters[$1] "," $2;  else clusters[$1] = $2} END { for (cluster in clusters) { print cluster, clusters[cluster] } }' > lag-dva_oneline
```

sample of one line of output data:

```
C36907R >dva152:268868-269825(+)::MAPQ=60,LENGTH=300,AS=119SeqStartagaacttttttgacggTCAGAGCTGTTGGACGGTGGAA…
```

4.3. Sort clusters into double, multi and poly-locus

```
cat lag-dva_oneline | awk -F">" '{if (NF<=3) print $0 >> "lag-dva_double"; else if (NF<11) print $0 >> "lag-dva_multi"; else print $0 >> "lag-dva_poly"}'
```

Three output files contain:

pairs of putative orthologous loci (lag-dva_double) - see section 5;

multiple entries (no more than 10) (lag-dva_multi) - see section 6;

more than 10 entries (lag-dva_poly) - not considered here (discarded).

The first two files (lag-dva_double and lag-dva_multi) are then analysed separately in sections 5 and 6.

5. Working with doubles

For each pair of aligned sequences, the presence of a SINE in one of them is tested, as well as the properties of both flanking sequences.

5.1. Splitting doubles into parts of size 100

```
awk 'NR%100==1{x="PART"++i;}{print > x}' lag-dva_double
```

5.2. Analyzing parts

The following code uses the custom script ComPair.sh which is provided in section 5.3:

```
for i in PART*; do
   Lines=$(awk 'END {print NR}' $i)
   clstrs=$i
   while [ $Lines -ge 1 ]
    do
     echo "Processing $Lines line"
     CurrCluster=$(awk -v line=$Lines 'NR == line' $clstrs)
     cName=$(echo $CurrCluster | awk '{print $1}')
     echo $CurrCluster |
      awk '{sub(/ /,"\n")}{gsub(/,>/,"\n>")}{gsub(/SeqStart/,"\n")} {print}' |
      awk 'NR==1 {a=$0} NR!=1 {if ($0 ~ ">") {print $0"::"a} else print}' | cat - $3 > "$cName".cl
     Lines=$(( $Lines - 1 ))
   done
   find -type f -name "*.cl" -print0 |
    xargs -0 -t -I % -P $(nproc) sh -c "mafft --op 5 --quiet '%' |
    seqkit seq -w 0 > '%.dbl'; ComPair.sh '%.dbl'; rm '%' '%.dbl'"
   rm $i
done
mv stat stat_doubles
```

The alignment of each cluster (pair of loci) in fasta format includes the Squam1 SINE consensus, and two loci from the **dva** and **lag** genomes.

Each alignment file name ends with ".dbl." followed by either "SINE", "PM" or "MP"

".dbl." indicates that the input alignment file contains 2 sequences (loci).

"SINE" - both loci contain SINE insertion.

"PM" - "plus-minus"; SINE insertion only in the first genome

"MP" - "minus-plus"; SINE insertion only in the second genome

The file "stat_doubles" contains statistics about the alignment computed by the ComPair.sh script.

Example of a line from stat_doubles:

./C8752R.cl.dbl SINE LF=300 FL=99 LFcp=298 RFlength=306 FR=98 RFcp=294 OneTwo=96 OneSINE=78 TwoSINE=79 SL=340 SO=278 ST=278

LF - length of the left (5') flank (including gaps)

FL - % identity in left (5') flanks

LFcp - number of identical nucleotides in left flanks

RFlength - length of the right flank (including gaps; can be trimmed if 3'-end contains unaligned overhang)

FR - % identity in right flanks

RFcp - number of identical nucleotides in right flanks

OneTwo - identity in SINE insertion sequence between genomes

OneSINE - identity between SINE in the first genome locus and SINE consensus

TwoSINE - identity between SINE in the second genome locus and SINE consensus

SL - number of identical bp between SINE insertion sequences in two genomes

SO - number of identical bp between SINE insertion in first genome locus and SINE consensus

ST - number of identical bp between SINE insertion in second genome locus and SINE consensus

The following values are used to determine the state of a pair of loci (“SINE”, “PM” or “MP”) using a script in section 5.3:

Quality check: alignment is considered poor if LF and RFlength are <=150, or the SINE is at the very beginning or end of the alignment, or if LFcp or RFcp or FL or FR are <=65; these alignments are excluded from analysis.

Assigninement to an alignment category: if OneTwo > 65 and SL > 100 and SO > 100 and ST > 100 then SINE insertion is present in both species and the alignment is marked as “SINE”; otherwise a locus may be polymorphic with respect to SINE insertion, if SO>ST then the alignment is marked as “PM”, otherwise the alignment is marked as “MP”.

It is important to note that the values of these parameters are determined by manual analysis of a representative sample of data for the two genomes in question; they are not universal and will probably need to be re-evaluated in another study depending on the SINE and the genomes used for comparison.

5.3. ComPair.sh

Custom script for analysis of multiple alignment of 3 sequences in fasta format: SINE consensus and two compared loci from studied genomes. It takes the alignment file name as the only argument ($1). The output file is classified as “plus-plus” (“SINE”) in both genomes at a given locus, or “plus-minus” (“PM”) or “minus-plus” (“MP”) for polymorphic loci.

```
# Each aligment of SINE-containing(?) loci is tested if it has
# 1) SINE in one genome and gap in other or SINE in both genomes
# 2) good left and right flanks.

# searching for consecutive gaps at both sides of SINE consensus in alignment
```

```
# find coordinates of SINE consensus relative to multiple alignment

echo $1
bank=$1
seqkit range -r -1:-1 -w 0 $bank | awk -F '[^-]+' 'NR!=1 {for (i=1; i<=NF; i++) if ($i != "") print length($i)}' > $bank.gaps # first and last values are lengths of 5' and 3' rows of consecutive gaps in SINE sequence
LF=$(awk 'NR==1' $bank.gaps) # right coordinate of the left flank
RFlength=$(awk 'END {print}' $bank.gaps) # length of the right flank
len=$(seqkit range -r -1:-1 -w 0 $bank | awk 'NR==2 { print length($0) }') # alignment length
RF=$((len-RFlength)) # left coordinate of the right flank
echo LF=$LF len=$len RF=$RF RFlength=$RFlength
rm $bank.gaps

#report problematic flanks length
if [[ "$LF" -le "150" ]]; then echo "Short FLANK. Trying to fix it"
    part=$(seqkit range -r -1:-1 -w 0 $bank | awk 'NR==2' | perl -pe 's/\p{L}([a-zA-Z]*)/"-" x (length($1)+1)/ei')
    awk -v part=$part 'NR<6 {print $0} END {print part}' $bank > $bank.temp
    mv $bank.temp $bank
    LF=$(seqkit range -r -1:-1 -w 0 $bank | awk -F '[^-]+' 'NR!=1 {print length($1)}') # recalculating new left flank coordinate
    echo new left flank $LF
fi
if [[ "$RFlength" -lt "150" ]]; then echo "Short FRANK"; mv $bank $bank.shortRF; status=$(echo "shortRF"); echo $bank $status >> stat; exit; fi

leftSINE=$(seqkit range -r -1:-1 -w 0 $bank | awk '/^[^-]/{if (NR==2) print "0"}')
if [[ "$leftSINE" == 0 ]]
       then
        echo "Skipping (leftSINE)"
        mv $bank $bank.lfSINE; status=$(echo "lfSINE")
echo $bank $status LF=$LF FL=$FL LFcp=$LFcp RFlength=$RFlength FR=$FR RFcp=$RFcp OneTwo=$OneTwo OneSINE=$OneSINE TwoSINE=$TwoSINE SL=$SL SO=$SO ST=$ST >> stat
        exit 111 # aborting search in this locus
fi

rightSINE=$(seqkit range -r -1:-1 -w 0 $bank | awk '{if (NR==2) {print substr($0,length($0),1)}}')
if [[ "$rightSINE" == "-" ]]
       then
        echo "right"
        else
        mv $bank $bank.rfSINE; status=$(echo "rfSINE")
        echo "Skipping (rightSINE)"
echo $bank $status LF=$LF FL=$FL LFcp=$LFcp RFlength=$RFlength FR=$FR RFcp=$RFcp OneTwo=$OneTwo OneSINE=$OneSINE TwoSINE=$TwoSINE SL=$SL SO=$SO ST=$ST >> stat
        exit 111 # aborting search in this locus
fi

#coordinates for cutting left flank, SINE and right flank
```

```
LFcut=$(( $LF + 1 ))
RFcut=$(( $RF + 1 ))

#gathering statistics. IMPORTANT: esl-alipid cannot process sequences <9bp

#FLANK
seqkit subseq -r 1:$LF -w 0 $bank | esl-alipid - | awk 'NR==2 {print $3,$4,"FLANK"}' > $bank.stats
LFcp=$(awk 'NR==1 {print $2}' $bank.stats) # number of identical nt in left flanks
#FRANK
seqkit subseq -r $RFcut:$len -w 0 $bank | esl-alipid - | awk 'NR==2 {print $3,$4,"FRANK"}' >> $bank.stats
RFcp=$(awk 'NR==2 {print $2}' $bank.stats) # number of identical nt in right flanks
#SINE
seqkit subseq -r $LFcut:$RF -w 0 $bank | esl-alipid - | awk 'NR==2 || NR==3 || NR==4 {print $3,"\n"$4}' >> $bank.stats # percentage
identity and number of identities

FL=$(awk -F. 'NR==1 {print $1}' $bank.stats) # identity in flanks
FR=$(awk -F. 'NR==2 {print $1}' $bank.stats) # identity in franks
OneTwo=$(awk -F. 'NR==3 {print $1}' $bank.stats) # identity in SINE between genomes
OneSINE=$(awk -F. 'NR==5 {print $1}' $bank.stats) # identity between first genome locus and SINE consensus
TwoSINE=$(awk -F. 'NR==7 {print $1}' $bank.stats) # identity between second genomelocus and SINE consensus
SL=$(awk -F. 'NR==4 {print $1}' $bank.stats) # number of identical bp between SINEs in two genomes
SO=$(awk -F. 'NR==6 {print $1}' $bank.stats) # number of identical bp between first genome locus and SINE consensus
ST=$(awk -F. 'NR==8 {print $1}' $bank.stats) # number of identical bp between second genome locus and SINE consensus

#check if SINE is + or - or bad, check if flank and/or frank is good

if [[ $LFcp -gt 65 && $FL -gt 65 ]] # check if FLANK is good
  then echo left flank looks good, next check SINE and right flank
        if [[ $OneTwo -gt 65 && $SL -gt 100 && $SO -gt 100 && $ST -gt 100 ]]
          then echo SINE in both, next check right flank
                if [[ $FR -gt 65 ]]
                  then echo right flank is good, homozygous copy; mv $bank $bank.SINE; status=$(echo "SINE")
                  else echo right flank bad, next try to fix it?; mv $bank $bank.badRF; status=$(echo "badRF")
                fi
          else echo SINE can be polymorphic, need to check right flank, preparing it first
                seqkit subseq -r $RFcut:$len -w 0 $bank > $bank.tocut
                cutRF=$(awk '!/^>/ { sub(/-*$/, ""); print}' $bank.tocut | awk 'NR<3 {print length($0)}' | sort -n | head -n 1)
#delete overhang at the tail of minus sequence
                  if [[ $cutRF -le 150 ]]
                        then echo Right flank too short; mv $bank $bank.shortRF; status=$(echo "shortRF"); rm $bank.tocut*
$bank*.fai $bank.stats
                        echo $bank $status LF=$LF FL=$FL LFcp=$LFcp RFlength=$RFlength FR=$FR RFcp=$RFcp
                OneTwo=$OneTwo OneSINE=$OneSINE TwoSINE=$TwoSINE SL=$SL SO=$SO ST=$ST >> stat; exit
                  fi
                seqkit subseq -r 1:$cutRF -w 0 $bank.tocut | esl-alipid - | awk 'NR==2 {print $3,$4,"FRANKcut"}' >> $bank.stats;
rm $bank.tocut*
```

```
                FRcut=$(awk -F. 'NR==9 {print $1}' $bank.stats) # identity in cut franks
                FRcp=$(awk 'NR==9 {print $2}' $bank.stats) # number of identical nt in cut franks
                if [[ $cutRF -gt 150 && $FRcut -gt 70 && $FRcp -gt 120 ]]
                  then echo right flank is good, heterozygous copy, next find which plus/minus
                        trim_right=$((RF+cutRF))
                        seqkit subseq -r 1:$trim_right -w 0 $bank > $bank.trim
                        if [[ $SO -gt $ST ]]
                          then
                                echo plus minus
                                mv $bank.trim $bank.PM; rm $bank; status=$(echo "PM")
                          else
                                echo minus plus
                                mv $bank.trim $bank.MP; rm $bank; status=$(echo "MP")
                        fi
                  else echo right flank bad, next try to fix it; mv $bank $bank.badRF; status=$(echo "badRF")
                fi
        fi
  else echo left flank bad $LFcp $LF $FL; mv $bank $bank.badLF; status=$(echo "badLF")
fi
echo $bank $status LF=$LF FL=$FL LFcp=$LFcp RFlength=$RFlength FR=$FR RFcp=$RFcp OneTwo=$OneTwo
OneSINE=$OneSINE TwoSINE=$TwoSINE SL=$SL SO=$SO ST=$ST >> stat
rm $bank.seqkit.fai $bank.stats
exit
```

The data regarding parts of an alignment (see section 5.2) are saved in an output “stat” file.

6. Working with multiples (clusters with more than two loci)

Clusters with more than two and less than 10 elements are evaluated to determine if there is only one “correct” pair of similar loci from two compared genomes and if other members of the cluster can be excluded from it. To do this, all sequences in a cluster are compared in a multiple alignment and the most similar pair is found based on an all-to-all similarity analysis. This pair is then analyzed as it was described above for doubles (section 5).

6.1. Split multiples into parts of size 100

```
awk 'NR%100==1{x="MULTIPART"++i;}{print > x}' lag-dva_multi
```

6.2. Convert each multipart cluster to fasta format and align to SINE consensus

```
# SINEname variable is derived from the name of a SINE used
```

```
for i in MULTIPART*; do
	Lines=$(awk 'END {print NR}' $i)
	clstrs=$i
	while [ $Lines -ge 1 ]
	do
	echo "Processing $Lines line"
	CurrCluster=$(awk -v line=$Lines 'NR == line' $clstrs)
	cName=$(echo $CurrCluster | awk '{print $1}')
	echo $CurrCluster | awk '{sub(/ /,"\n")}{gsub(/,>/,"\n>")}{gsub(/SeqStart/,"\n")} {print}' | awk 'NR==1 {a=$0} NR!=1 {if ($0 ~
">") {print $0"::"a} else print}' > "$cName".cl
	cat $cName.cl $3 > $cName.clu
	rm $cName.cl
	Lines=$(( $Lines - 1 ))
	done
	find -type f -name "*.clu" -print0 | xargs -0 -t -I % -P $(nproc) mafft --op 5 --quiet % | seqkit seq -w 0 > multemp
	awk  -v SINEname=$SINEname '{print}f{print "";f=0} {if ($0~SINEname) f=1}' multemp > $clstrs.mul
	rm *.clu multemp
done
```

Each cluster is saved as a multiple alignment in fasta format as a *.mul file.

6.3. Finding the best pair of sequences from different genomes in each cluster

```
# SINEname variable is derived from the name of a SINE used
# sp1 and sp2 variables are the names of two genomes
# ComPair.sh is a script described in section 5.3

find -type f -print -name "*.mul" -print0 -exec sh -c "esl-alipid {} |
 awk -v sp1=$sp1 -v sp2=$sp2 -v SINEname=$SINEname 'NR>1 (sp1=substr(\$1,1,3)) (sp2=substr(\$2,1,3)) {if (sp1!=sp2 && \$0
!~SINEname) print}' |
 LC_ALL=C sort -r -k 3 -g |
 awk  -v SINEname=$SINEname 'NR==1 {  print \$1,\$2,SINEname,"\n" }' | sed 's/\s\+/\n/g' >  '{}.list'
 seqkit grep -f '{}.list' '{}' > '{}.doub'; rm '{}.list'" \;

find -type f -name "*.doub" -print0 | xargs -0 -t -I % -P $(nproc) sh -c "mafft --op 5 --quiet '%' | seqkit seq -w 0 > '%.doubles';
ComPair.sh '%.doubles'; rm '%' stat"

for i in *.mul.*bad*F *.mul.*shortRF *.mul.*.lfSINE *.mul.*.rfSINE; do rm $i "${i//.doub.doubles*/}" ; done
```

Each cluster is reduced to a pair of most similar sequences and assigned a “plus-minus”, “minus-plus” or “plus-plus” state as described in section 5.3.

6.4. Additional check for good sequences in each cluster by right flank similarity in right flanks and maximum total alignment length

```
for i in *.mul.*.MP *.mul.*.PM *.mul.*.SINE; do [ -f "$i" ] || continue;
 name1=$(awk 'NR==1{print substr($1,2)}' "$i");
 name2=$(awk 'NR==3{print substr($1,2)}' "$i");
echo name1=$name1 name2=$name2;
 SINEend=$(seqkit range -r -1:-1 -w 0 "${i//.doub.doubles*/}" | awk -F '[^-]+' 'END {print length($NF)}');
 ALIGNMENTend=$(seqkit range -r -1:-1 -w 0 "${i//.doub.doubles*/}" | awk 'END {print length}');
 CUT=$((ALIGNMENTend-SINEend+1));
awk -v CUT=$CUT -v ALIGNMENTend=$ALIGNMENTend 'BEGIN{RS=">";FS="\n"}NR>1{seq="";
for (i=2;i<=NF;i++) seq=seq""$i; print ">"$1"\n"substr(seq,CUT,ALIGNMENTend-CUT)}' "${i//.doub.doubles*/}" |
 esl-alipid - | awk -v name1="$name1" -v name2="$name2" -v SINEname=$SINEname '$0!~SINEname {if ($1==name1 ||
$1==name2) print}' |
 awk '{if ($3>40.00) print $1"\n"$2}' |
 awk -v name1="$name1" -v name2="$name2" '{print}END {print name1"\n"name2}' | awk '!seen[$0]++' > $i.list; ListSize=$(awk
'END{print NR}' $i.list)
if [[ $ListSize -eq 0 ]]; then mv $i $i."sel.aligned"; rm $i.list
 else
 seqkit grep -f $i.list -w 0 "${i//.doub.doubles*/}" > $i.SelSeq; rm $i.list
 seqkit range -r -1:-1 -w 0 $i > $i.SelSINE; cat $i.SelSeq $i.SelSINE > $i.selected;
 rm $i "${i//.doub.doubles*/}" $i.SelSeq $i.SelSINE;
fi
done
```

```
for k in *.selected; do Count=$(grep -c ">" $k); if [[ $Count -ne "3" ]]; then rm $k; fi; done

find -type f -name "*.selected" -print0 |
 xargs -0 -t -I % -P $(nproc) sh -c "mafft --op 5 --quiet '%' |
 seqkit seq -w 0 > '%.sel.aligned'; ComPair.sh '%.sel.aligned'; rm '%'"

for i in *.mul.*.MP *.mul.*.PM *.mul.*.SINE; do Len=$(awk '{if (NR==2) print length}' $i);
 if [[ $Len -ge 1600 ]]
    then rm $i
 fi
done

mv stat stat_multi_"$sp1"-"$sp2"
```

Only pairs of loci with reliable right flanks and expected alignment length are selected and classified.

Statistics

```
awk -F"./" '{print $2}' stat_multi_"$sp1"-"$sp2" stat_doubles_"$sp1"-"$sp2" | awk '{if ($2=="PM" || $2=="MP" || $2=="SINE") print}' |
awk -F. '{print $1,$0}' > statdata

for i in *.cl.*.PM *.cl.*.MP *.cl.*.SINE; do awk -F:: '{if (NR==1 || NR==3) print FILENAME,$1}' $i |  awk -F. '{print $1,$NF}' | awk
'{sub(/>/,"")} {print $1,$3}' >> statcoords; done

cat statcoords statdata | sort | awk '$1 in a {print a[$1]"\n"$0; next} {a[$1]=$0}' | sort | uniq > statcomb

awk '$1 in a {print a[$1]"\n"$2; next} {a[$1]=$0}' statcomb | awk '!seen[$0]++' | tac | awk '{if (NF==1) printf $0" "; else print }' >
statbed_"$sp1"-"$sp2"

while read col1 col2 rest; do echo $col2 $col1 $rest; done < statbed_"$sp1"-"$sp2" > statbed_"$sp2"-"$sp1"_reversed

awk '{sub(/\(/,"\t0\t0\t")}{sub(/:/,"\t")}{sub(/)/,"\t")}{sub(/-/,"\t")}'1 statbed_"$sp1"-"$sp2" > statbed_"$sp1"-"$sp2".bed

awk '{sub(/\(/,"\t0\t0\t")}{sub(/:/,"\t")}{sub(/)/,"\t")}{sub(/-/,"\t")}'1 statbed_"$sp2"-"$sp1"_reversed > statbed_"$sp2"-"$sp1".bed

awk '{if (NF>3) print $3}' statcomb | sort | uniq -c > output_"$sp1"-"$sp2".txt
```

Output

Alignments of all pairs of loci with a SINE insertion in at least one genome are saved as separate files.
The following data is provided in a result table for each genome:

1. genomic coordinates of a locus in one genome
2. corresponding coordinates of a locus in the second genome
3. cluster name
4. alignment file name
5. type of loci - “plus-minus”, “minus-plus” or “plus-plus”
6. whether a locus was initially identified as a double or as multi-matching
7. a number of statistical pamateters of loci alignment as described in section 5.2.

Example:
lag10:6945193-6946164(-) dva323:190012-190969(+) C102989R C102989R.cl.dbl PM LF=313 FL=95 LFcp=285 RFlength=675 FR=91 RFcp=275 OneTwo=0 OneSINE=89 TwoSINE=0 SL=0 SO=318 ST=0

Description: a locus in the lag genome at coordinates lag10:6945193-6946164 is found to be significantly similar in flanking sequences to a locus dva323:190012-190969(+) in a target genome, with SINE insertion only in dra (and absence in lag). Left flanks are 313bp long and 95% identical (with 285 identical nt). Right flanks start at 675bp, with 275 identical nt and 91% similarity. First sequence (from dva genome) contains SINE insertion with 89% similarity to consensus and length 275nt; and other sequence (from lag genome) has a gap.
