# Supplementary material for: Interspecific Comparison of Orthologous Short Interspersed Elements Loci Using Whole-Genome Data
Source: Genes (Basel). 2023 Nov 17;14(11):2089. doi: 10.3390/genes14112089 (PMC10670947; doi:10.3390/genes14112089)

Searching for SINE loci in both genomes

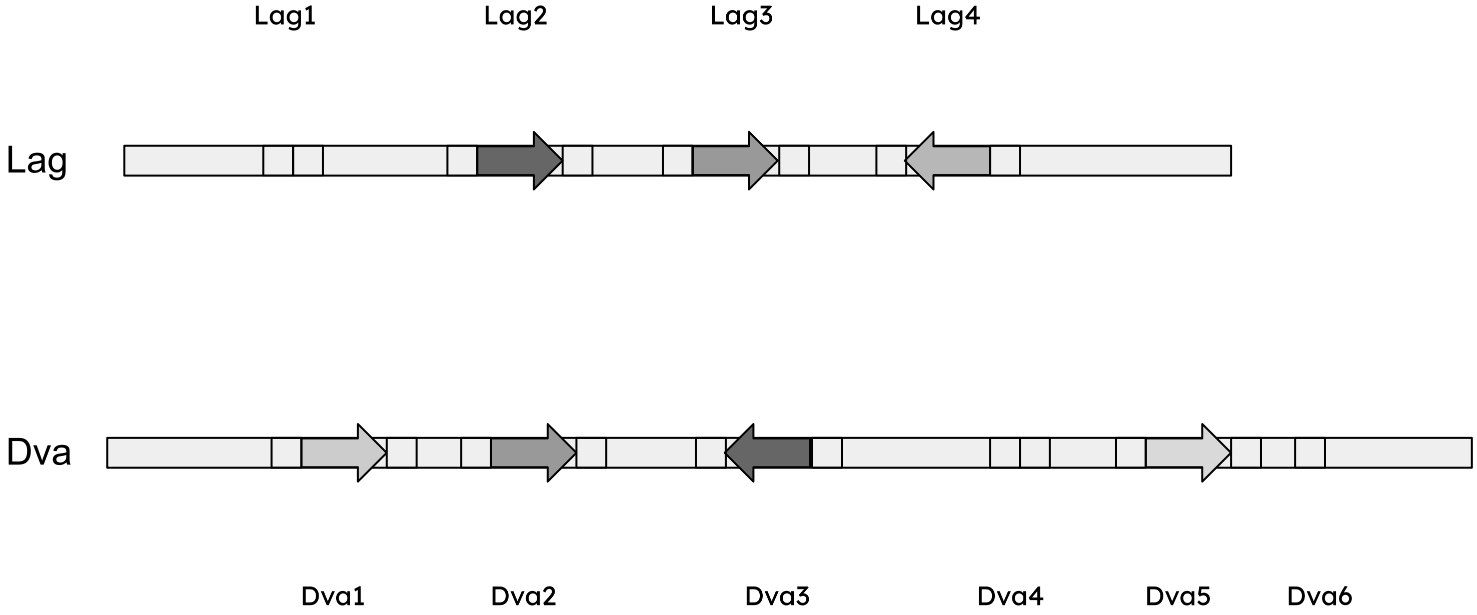

Gray arrow boxes are SINE insertions, adjacent boxes are flanking regions (or their putative orthologs if a SINE is absent in one of the genomes) that will be used in the analysis.
Loci names are given above or below each locus.

Identifying and extracting left 300-bp flanks from Lag

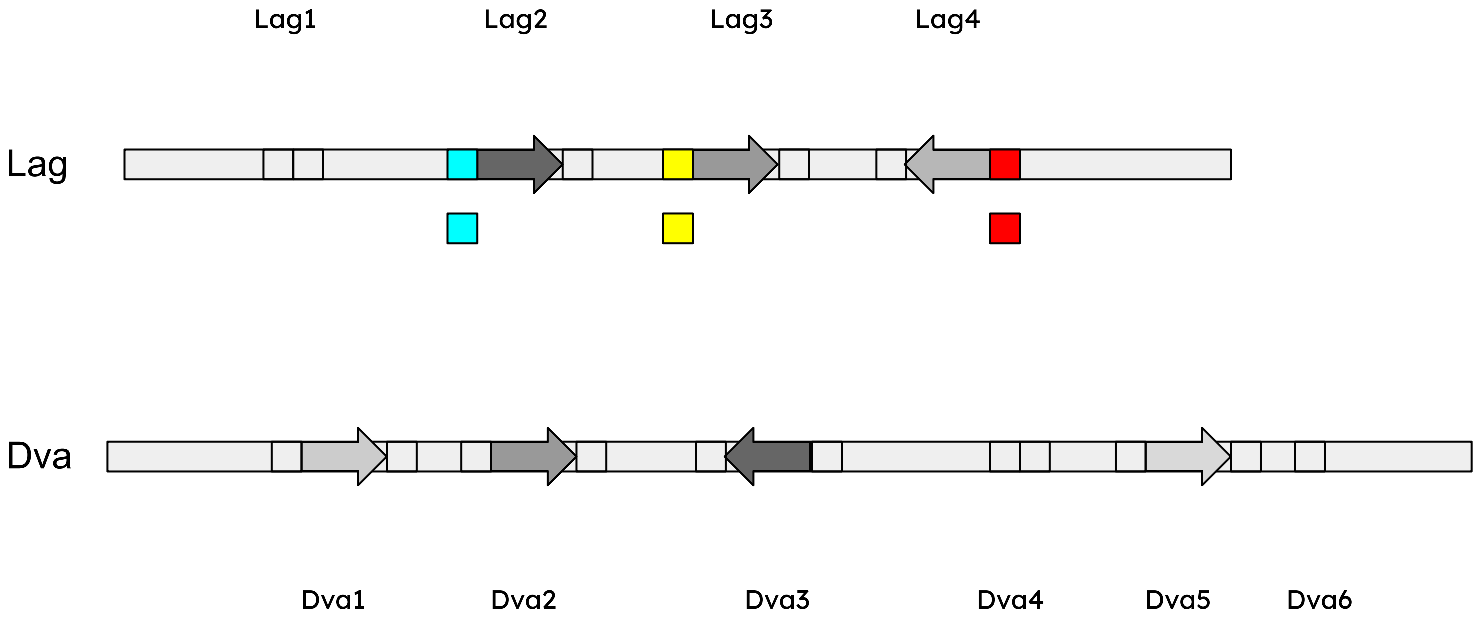

Coloured boxes indicate left flanking sequences for SINE copies in lag genome.

Mapping Lag flanks on Dva genome using BWA MEM

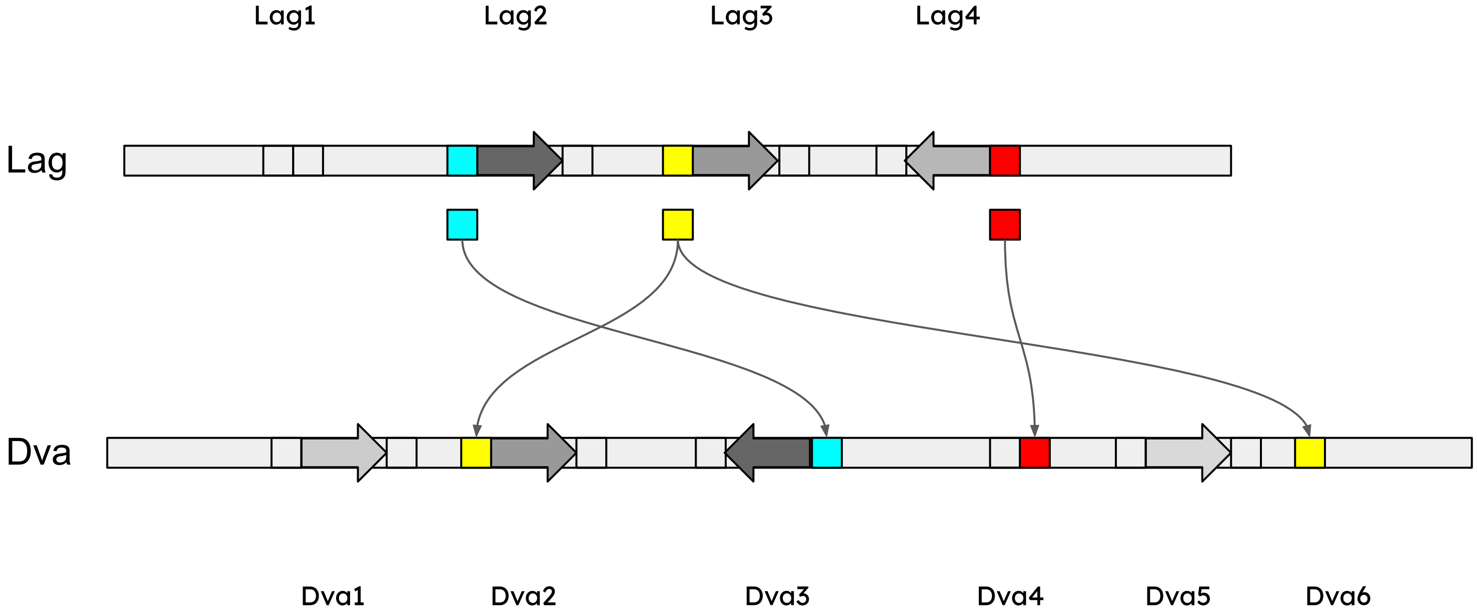

Sequences of left flanks from Lag are mapped on Dva genome. Arrows indicate direction of mapping.
One query may find more than one match in the target genome (yellow boxes).

Extracting coordinates of target Dva flanks

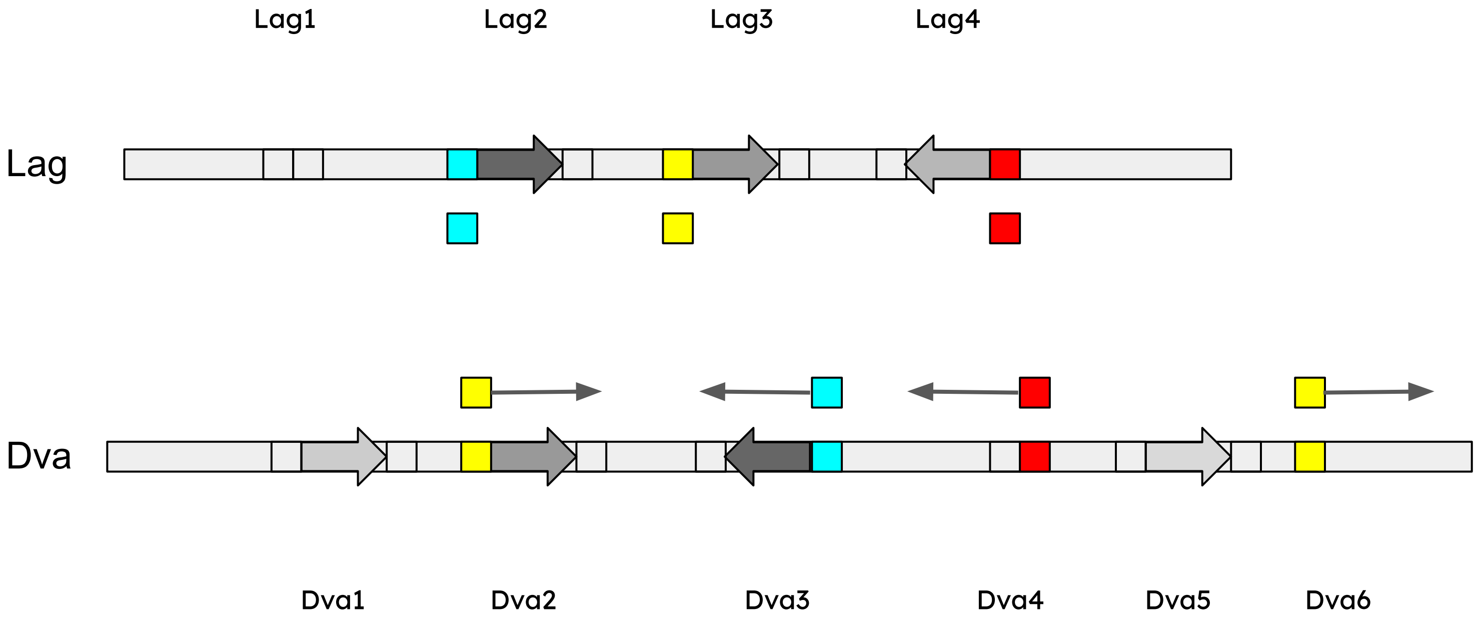

Arrows indicate a direction of an extension of coordinates to cover left flank+ SINE+right flank.

Extending Dva flanks

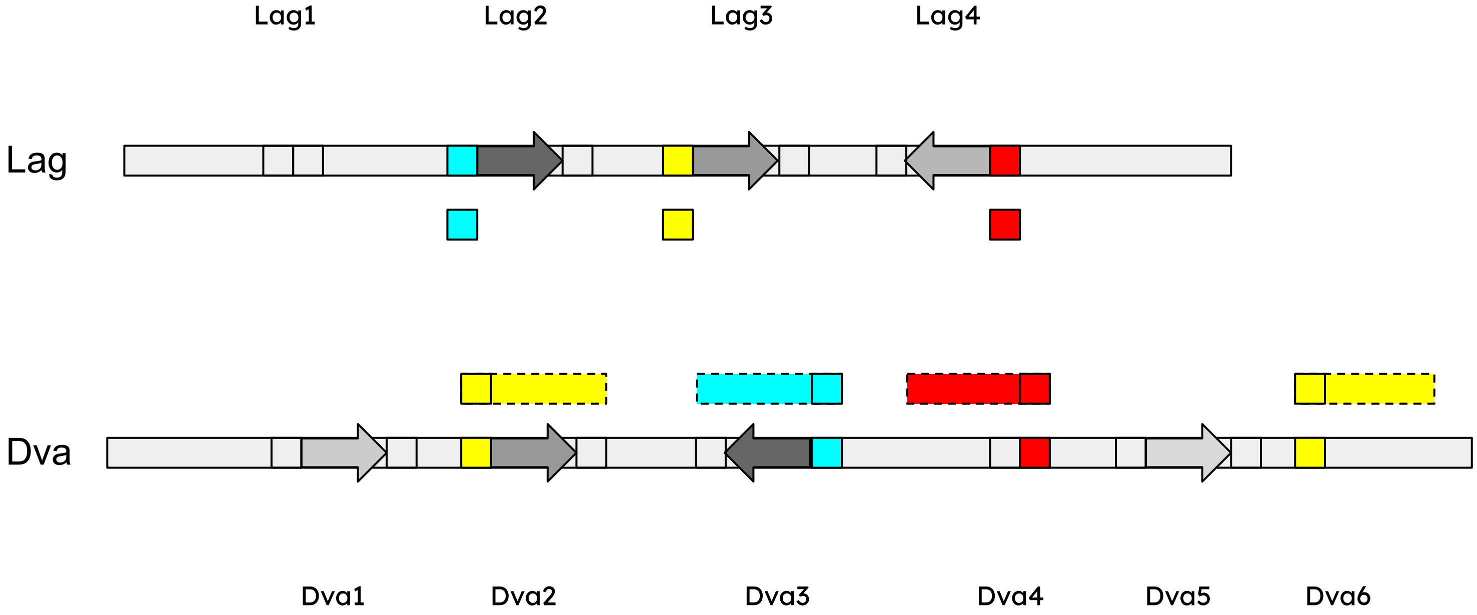

Sequences of extended Dva loci are extracted (solid+dashed lines around coloured boxed above Dva loci).

Extending Lag flanks

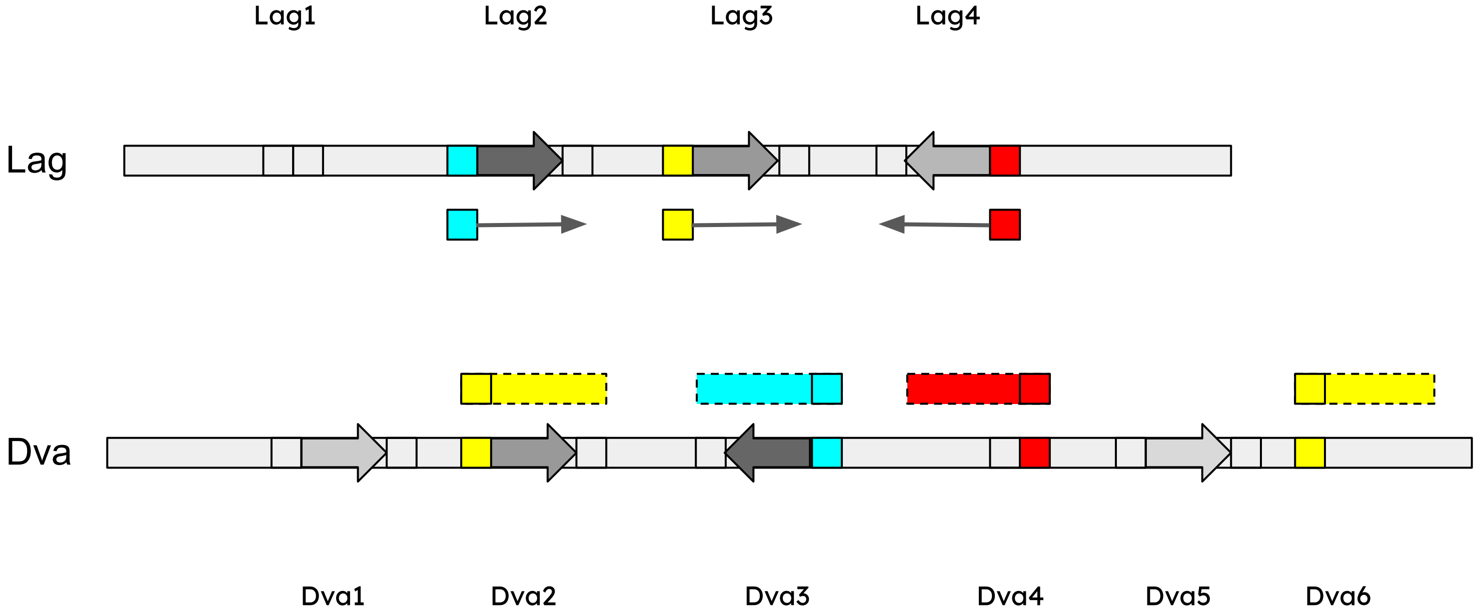

As in previous step, sequences of Lag loci are extracted for pairwise alignment with Dva corresponding loci.

Lag->Dva pairs of loci are ready for comparison in doubles or multiples

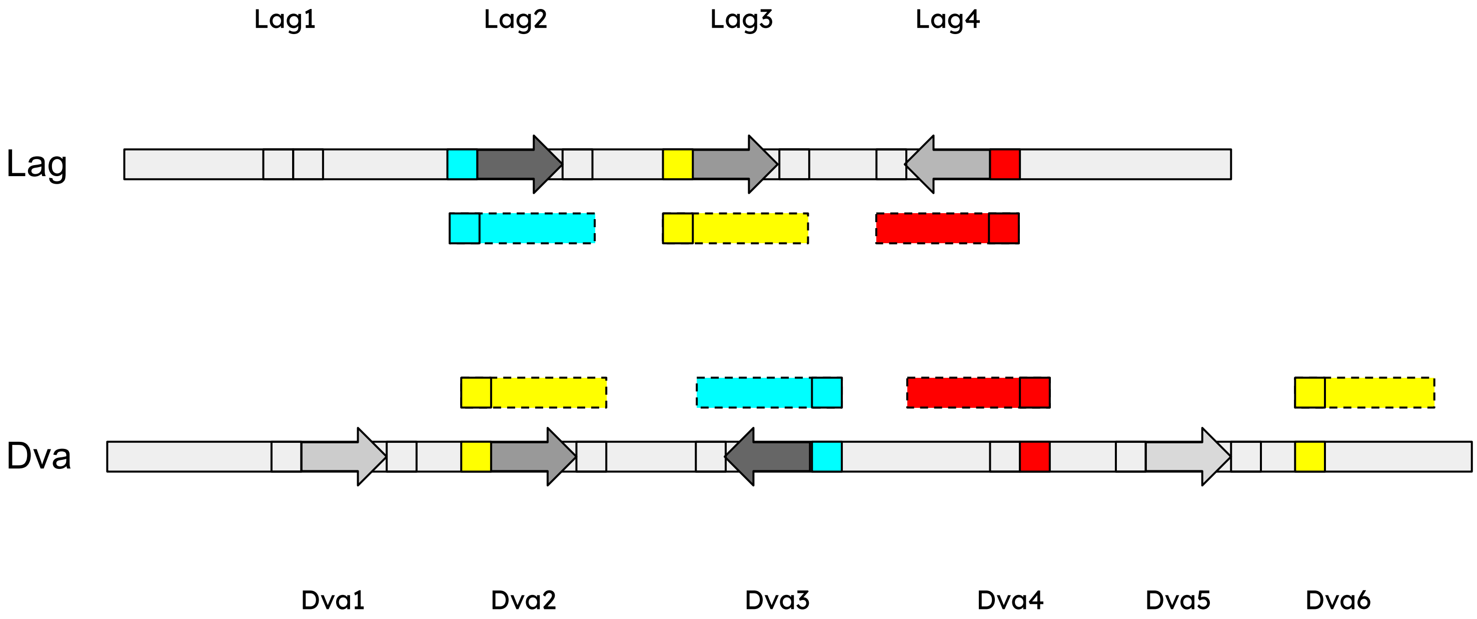

A set of loci from first direction of mapping is ready for analysis of flanks and SINE insertion. For Dva loci, there is no information about presence of SINE in them yet.

Identifying and extracting left 300-bp flanks from Dva

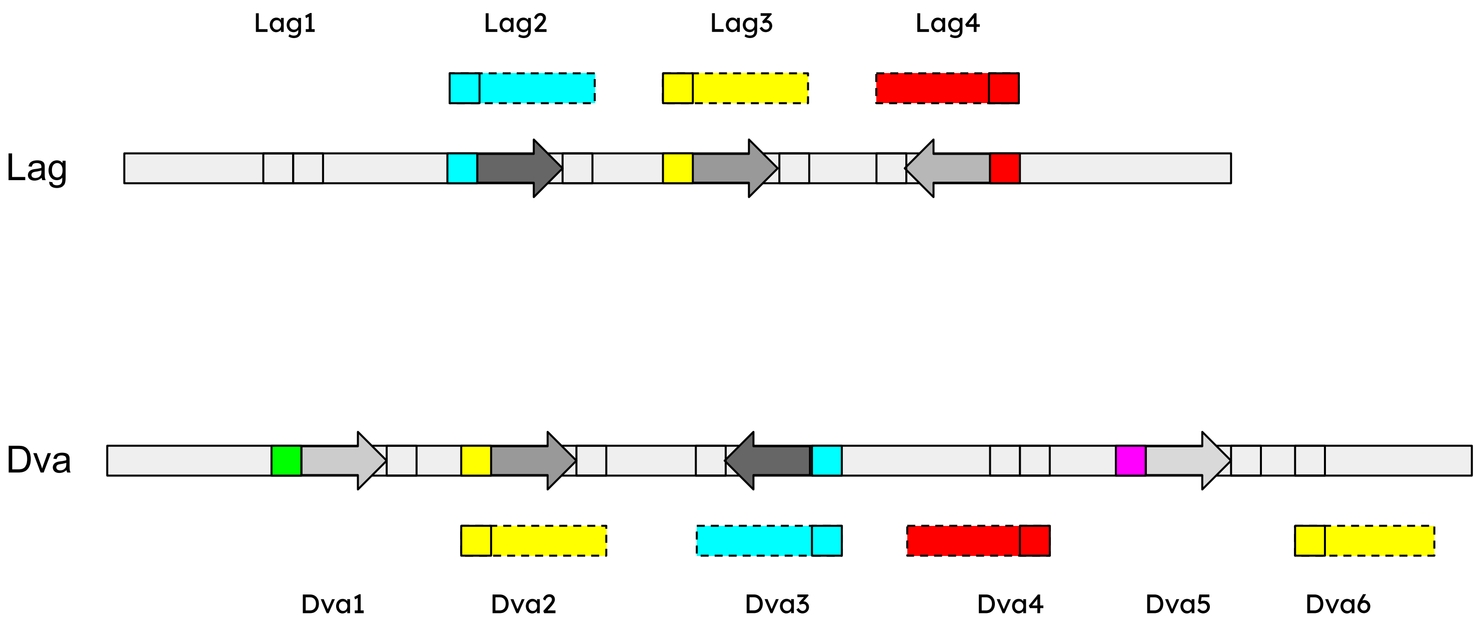

Left flanks in Dva genome are determined (coloured boxes on the Dva genome rectangle).
Sequences from previous steps are saved for later analysis (shown above the Lag and below the Dva genomes).

Identifying and extracting left 300-bp flanks from Dva

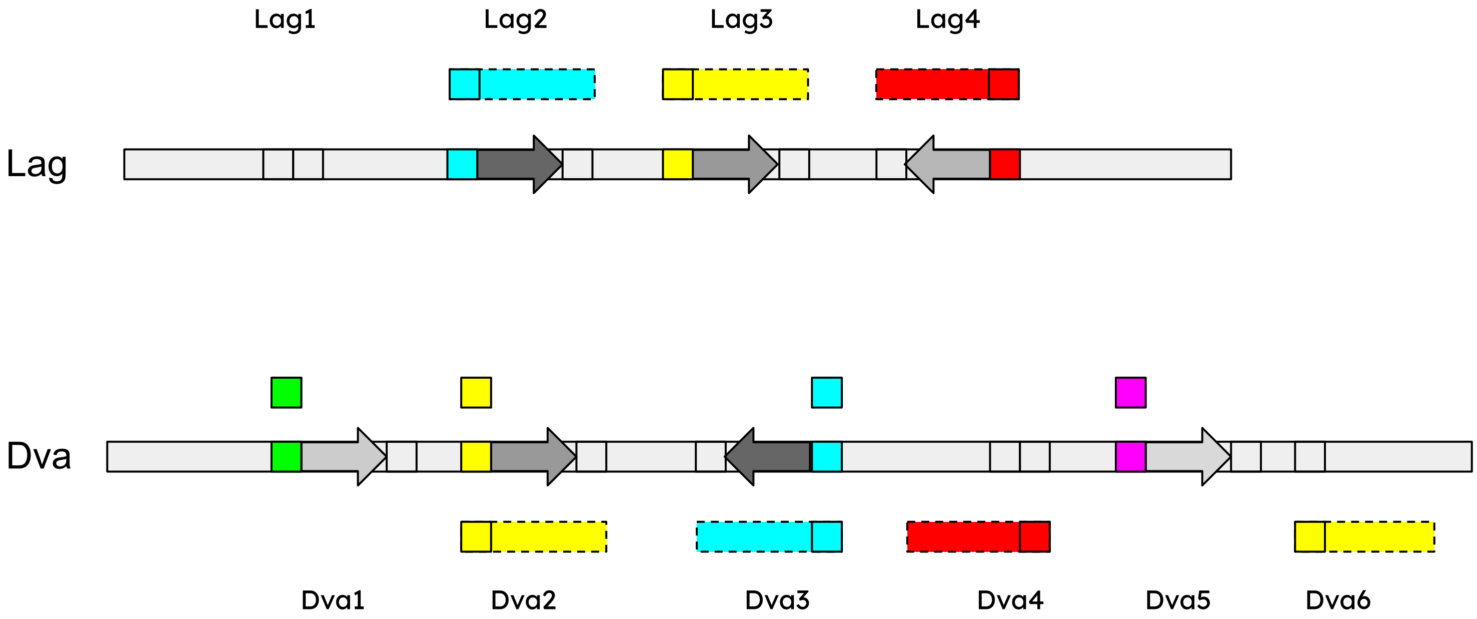

Left flanks in Dva genome are collected for mapping on Lag genome.
Resulting sequences in this direction of mapping (Dva->Lag) are shown between grey genome bars.

Mapping Dva left flanks on Lag genome

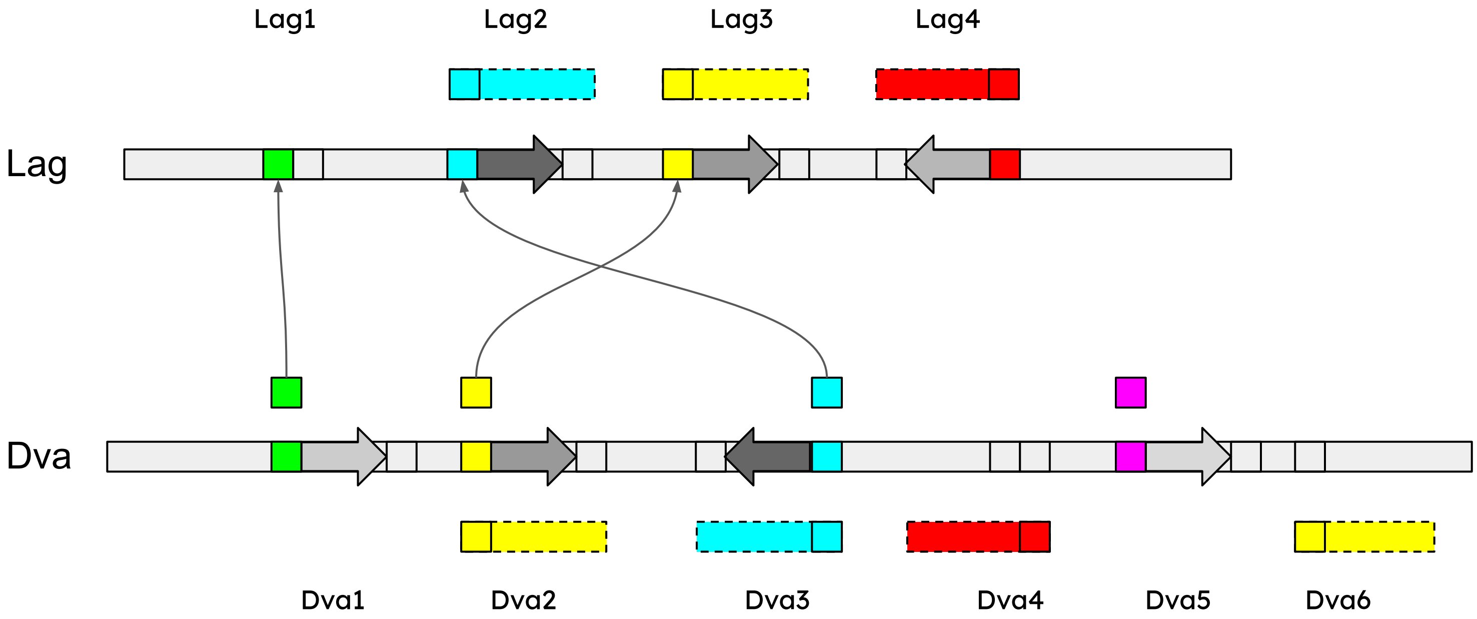

Successfully mapped flanks are connected with arrows with their targets in Lag genome.

Extracting coordinates of matches in Lag genome

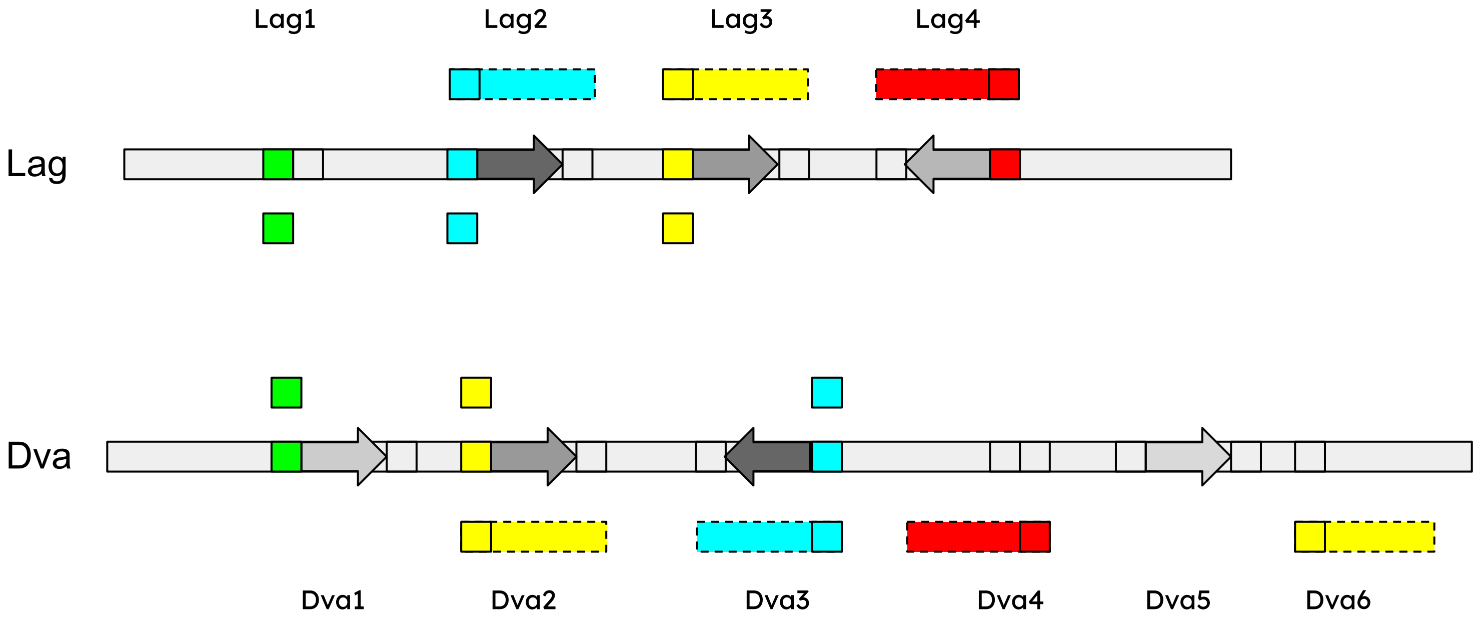

Dva5 locus is excluded from analysis as non-matching in Lag genome.

Extending Lag and Dva flanks

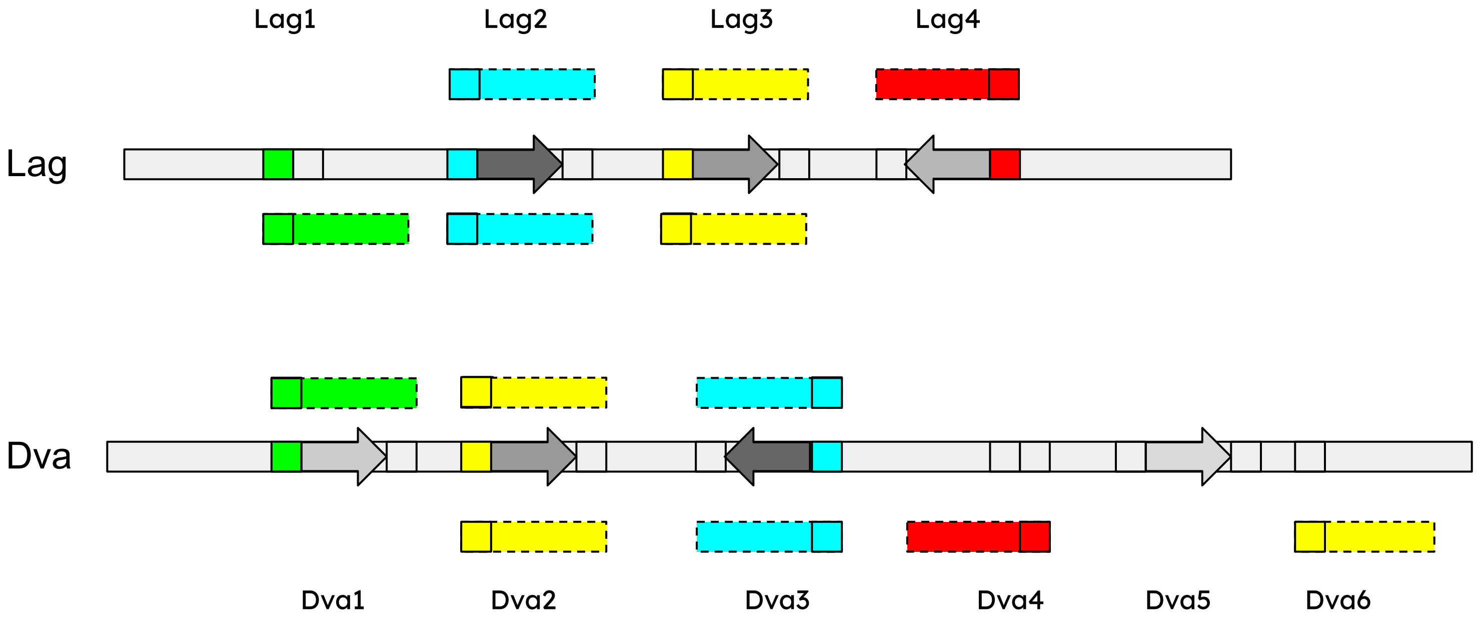

Pairs of loci with sequences matching to left flanks from Dva are extracted from both genomes.

Actual relations of Lag and Dva loci based on 2-way comparison of their left flanks

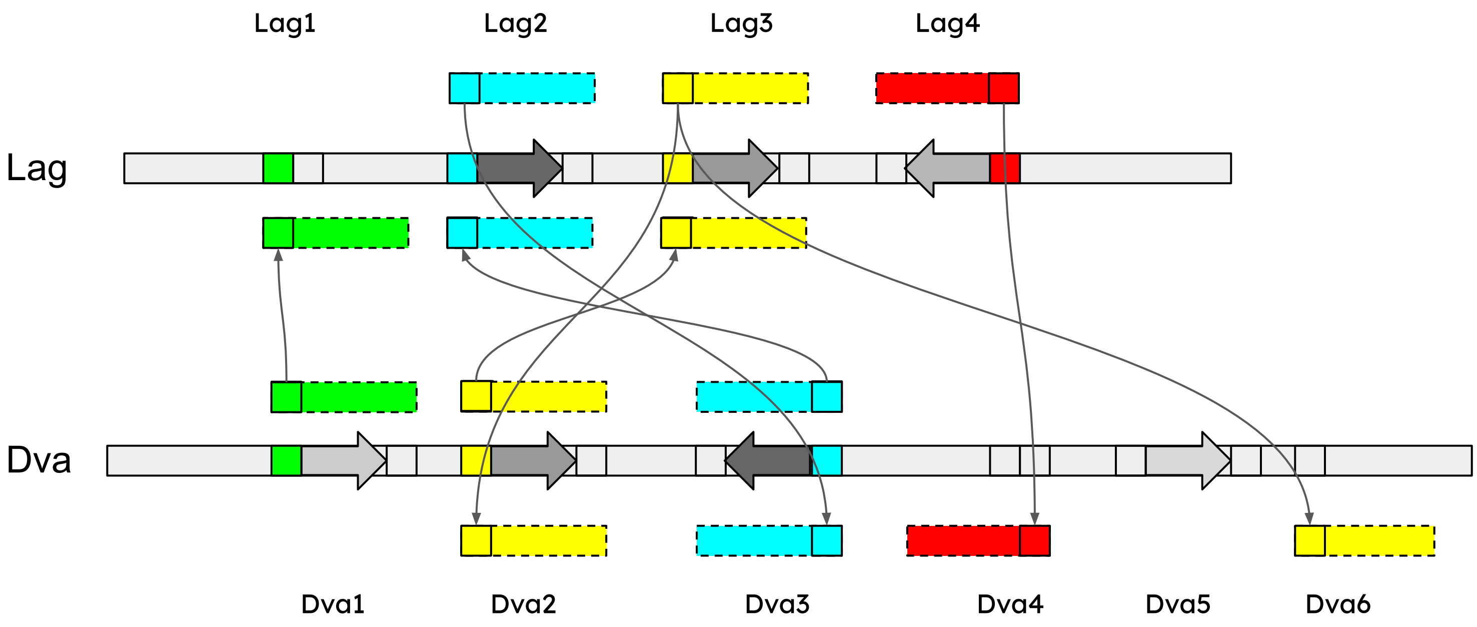

An overview of interconnections between loci (indicated by arrows, from query to target).

Assigning names for each loci

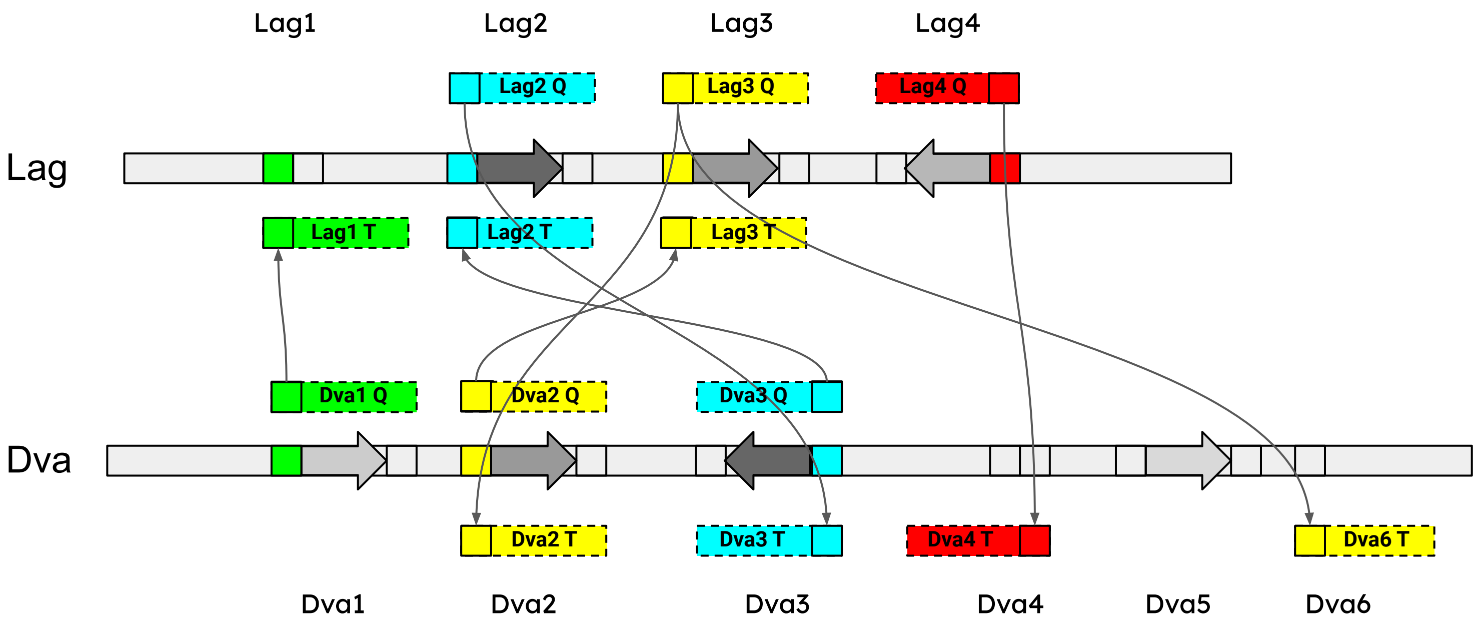

“Q” in locus name indicates query genome, “T” indicates target. Coordinates of “T” and “Q” loci with the same name of a locus have overlapping coordinates.

Grouping loci in matching pairs (doubles or multiples)

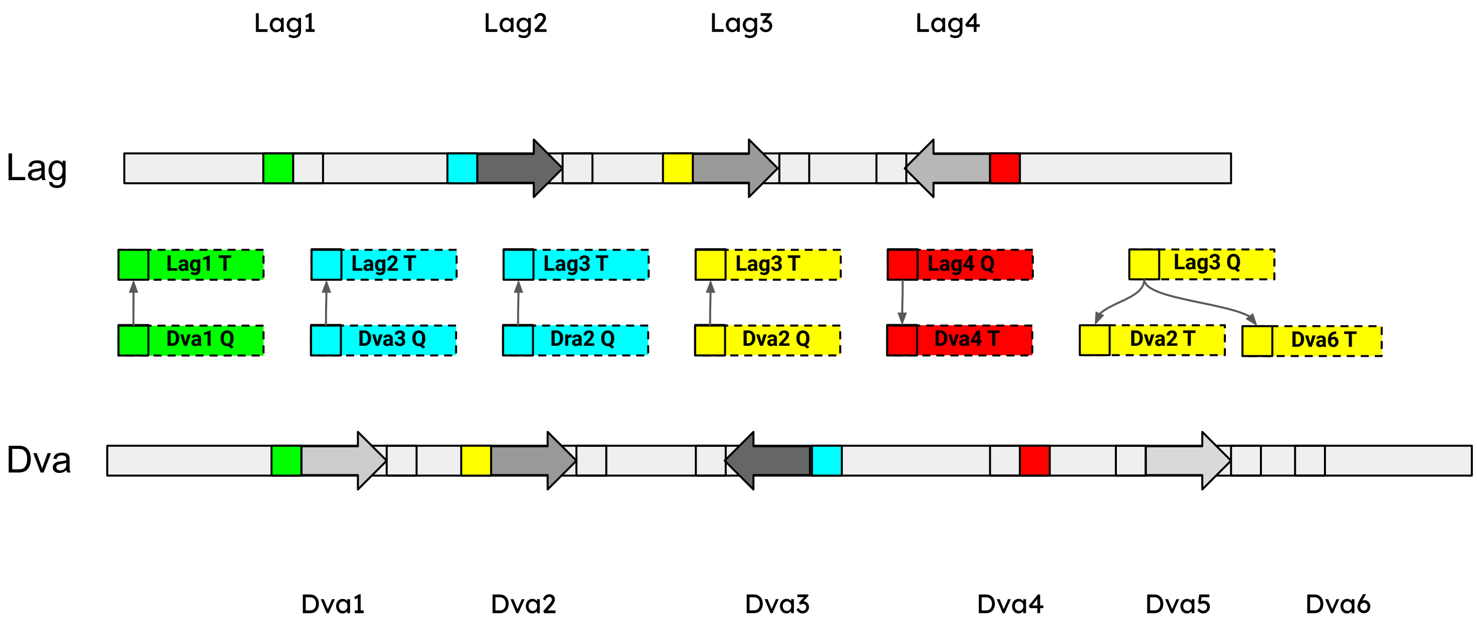

Pairs of loci sequences are shown in the middle.
Pair of green loci is found only in one direction of screening and represents possible polymorphic one with respect to SINE insertion. Red loci exemplifies same situation but in another direction of mapping.
Blue loci (a single pair of loci represented by four sequences) possibly indicates a SINE insertion in both genomes.
Yellow loci represent one of many possible variants of multiple matches.

Separate analysis of doubles and multi-matching loci.

Doubles are reduced (merged) to a pair of sequences when appropriate.
In a cluster of blue loci (Cluster2), the sequences Lag2T+Lag2Q and Dva3Q+Dva2Q are merged on the basis of their overlapping genomic coordinates.

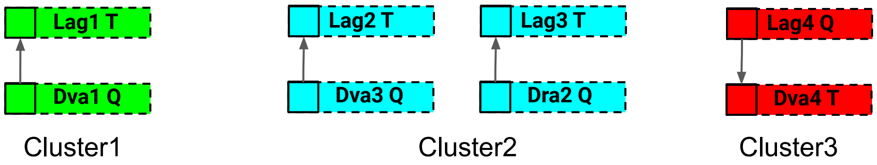

Superclusters are merged by genomic coordinates for each genome, and the number of remaining loci is counted. Clusters with 10+ loci are discarded. Clusters with less than 10 loci are checked if a distinct pair of most similar sequences between genomes can be found; in which case a supercluster is reduced to a double and is later analysed as it was described for doubles.

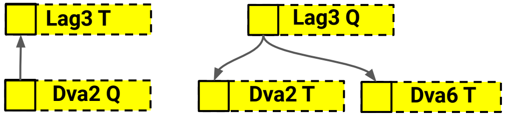

Different colours correspond to different clusters (in a case of doubles) or superclusters (in a case of multi-loci).

Analysis of multi-matching loci.

In the case of the yellow supercluster, if Lag3 has a higher similarity to Dva2 than to Dva6 locus, then Dva6 locus is excluded from this supercluster. The sequences of Lag3 and Dva2 are then analysed as a double.

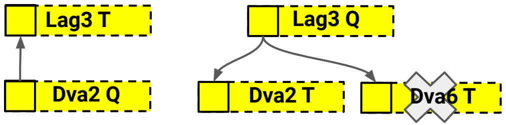

Analyzing loci for presence of a SINE and a matching right flank sequence

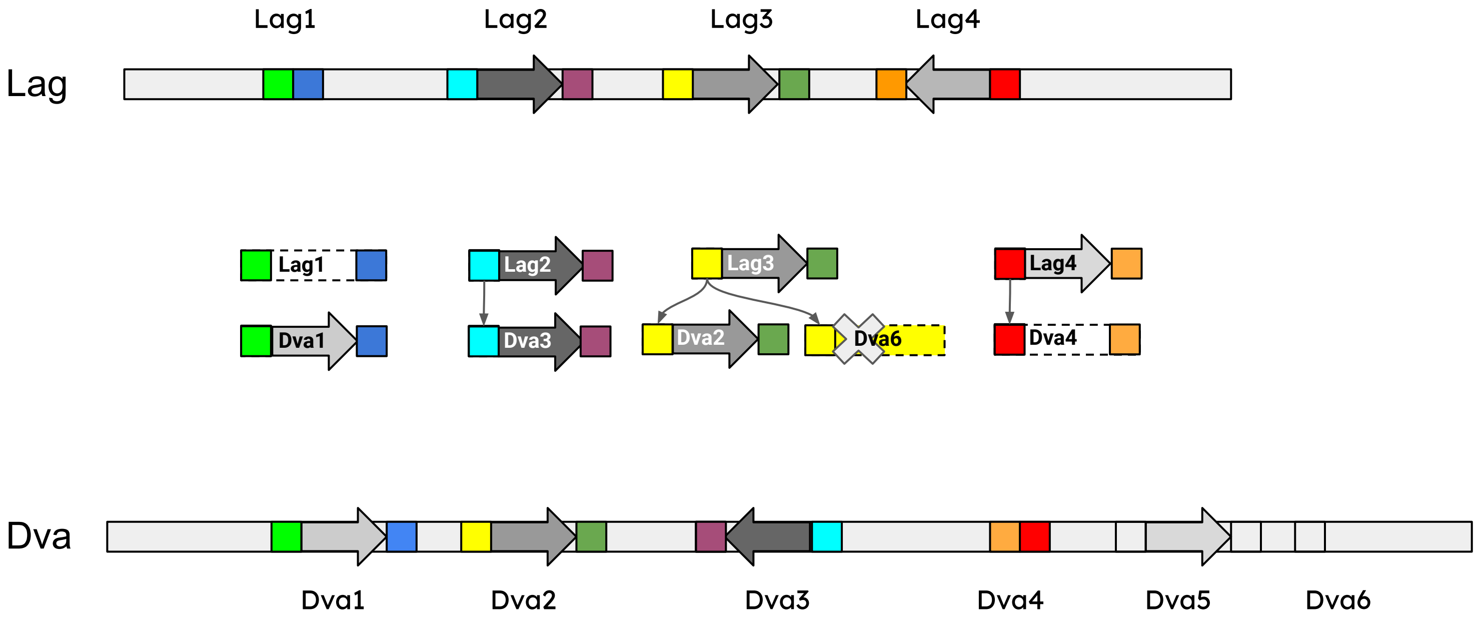

In-depth analysis of both flanks and putative SINE region in all pairs of loci.

Assignment of a “plus” or “minus” status for each locus.

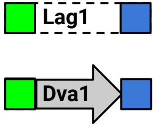

“Minus-plus” locus with SINE insertion only in Dva

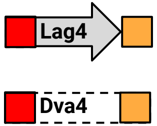

“Plus-mins” locus with SINE insertion only in Lag

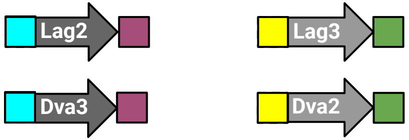

“Plus-plus” loci with SINE insertion in both genomes.
Lag3-Dva2 was initially a multi-matching supercluster.

Parameters of a pair of loci aligned with SINE family consensus.

Length (with gaps), number of identical nucleotides and % similarity are reported for:

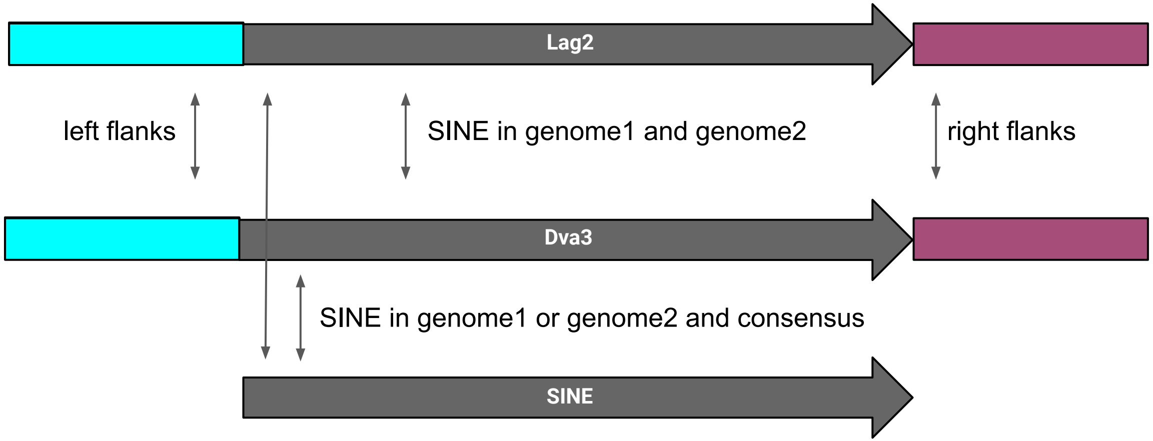

Supplement: Supplementary file 1 [file genes-14-02089-s001.zip › Supplemental File 4.pdf]
